# High-Defect-Density Graphite for Superior-Performance Aluminum-Ion Batteries with Ultra-Fast Charging and Stable Long Life

**DOI:** 10.1007/s40820-021-00698-0

**Published:** 2021-08-09

**Authors:** Jisu Kim, Michael Ruby Raj, Gibaek Lee

**Affiliations:** Advanced Energy Materials Design Lab, School of Chemical Engineering, Yeungnam University, Gyeongsan, 38541 Republic of Korea

**Keywords:** Surface modification, Etched graphite, Cathode materials, Energy storage, Aluminum-ion batteries

## Abstract

Rechargeable aluminum-ion batteries (AIBs) are a new generation of low-cost and large-scale electrical energy storage systems. However, AIBs suffer from a lack of reliable cathode materials with insufficient intercalation sites, poor ion-conducting channels, and poor diffusion dynamics of large chloroaluminate anions (AlCl_4_^−^ and Al_2_Cl_7_^−^). To address these issues, surface-modified graphitic carbon materials [i.e., acid-treated expanded graphite (AEG) and base-etched graphite (BEG)] are developed as novel cathode materials for ultra-fast chargeable AIBs. AEG has more turbostratically ordered structure covered with abundant micro- to nano-sized pores on the surface structure and expanded interlayer distance (*d*_002_ = 0.3371 nm) realized by surface treatment of pristine graphite with acidic media, which can be accelerated the diffusion dynamics and efficient AlCl_4_^−^ ions (de)-intercalation kinetics. The AIB system employing AEG exhibits a specific capacity of 88.6 mAh g^−1^ (4 A g^−1^) and ~ 80 mAh g^−1^ at an ultra-high current rate of 10 A g^−1^ (~ 99.1% over 10,000 cycles). BEG treated with KOH solution possesses the turbostratically disordered structure with high density of defective sites and largely expanded *d*-spacing (*d*_002_ = 0.3384 nm) for attracting and uptaking more AlCl_4_^−^ ions with relatively shorter penetration depth. Impressively, the AIB system based on the BEG cathode delivers a high specific capacity of 110 mAh g^−1^ (4 A g^−1^) and ~ 91 mAh g^−1^ (~ 99.9% over 10,000 cycles at 10 A g^−1^). Moreover, the BEG cell has high energy and power densities of 247 Wh kg^−1^ and 44.5 kW kg^−1^. This performance is one of the best among the AIB graphitic carbon materials reported for chloroaluminate anions storage performance. This finding provides great significance for the further development of rechargeable AIBs with high energy, high power density, and exceptionally long life.

**Supplementary Information:**

The online version contains supplementary material available at 10.1007/s40820-021-00698-0.

## Highlights

- Surface-modified graphite (acid-treated expanded graphite (AEG)/base-etched graphite (BEG)) displays an abundant micro- to nano-sized pores/defects and exposed edge graphitic site.
- AEG and BEG exhibit high specific capacity, rate capability and cyclic stability compared with pristine graphite.
- High defect density of BEG electrode exhibits profound effect on the chloroaluminate anions intercalation kinetics.

**Supplementary Information:**

The online version contains supplementary material available at 10.1007/s40820-021-00698-0.

## Graphical abstract

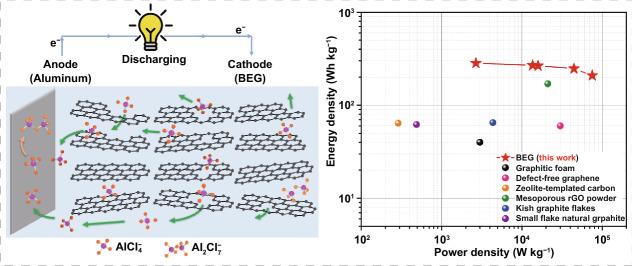

## Introduction

Rechargeable aluminum-ion battery (AIB)-based energy storage devices have significant advantages such as low material cost, high abundance, well-defined charge–discharge plateaus, high specific energy, long-term cycle life, and ease of handling in ambient environments [1–3]. Moreover, the safety of metallic aluminum (Al) anodes follows a three-electron-transfer redox reaction owing to its trivalent (Al^3+^) nature (i.e., the strong Coulombic effect of Al^3+^ ions), which leads to a high theoretical gravimetric capacity (~ 2980 mAh g^−1^) and approximately fourfold volumetric capacity (~ 8046 mAh cm^–3^) compared with those of lithium anodes (~ 2080 mAh cm^–3^) [4–6]. In early efforts, different types of cathode materials, such as carbon materials [7–11], metal oxides (V_2_O_5_, VO_2_, and TiO_2_) [12, 13], metal sulfides and selenides [14–16], and conducting polymers [17], have been widely applied to rechargeable AIBs. However, their practical applications were substantially limited due to inadequate cathodic performance such as low specific capacity, low charge–discharge voltage plateaus, and shorter cycle life. Recent studies have utilized various types of graphitic carbon materials, including defect-free graphene aerogel [18], porous 3D graphene foam (GNHPG) [19], graphene microflowers [20], natural graphite flakes [21], small graphite nanoflakes [22], zeolite-templated carbon [23], edge-rich graphene paper [24], carbon nanoscrolls [25], graphene nanoplatelets (GNPs) [26, 27], pyrolytic graphite [28], and 3H3C graphene films [2]. In addition, conjugated polymers [29] and triangular macrocycles [30] have been extensively explored as cathode materials for chloroaluminate anions/cation storage in AIBs. Among them, graphitic carbon materials are highly promising owing to their favorable discharge capacity due to the reversible capability of chloroaluminate ions (de)intercalation, ultra-fast charging rate capability, and ultra-stable cycling stability using an ionic liquid (IL) as the AIB electrolyte (consisting of 1-ethyl-3-methylimidazolium chloride ([EMIm]Cl) and aluminum chloride (AlCl_3_) in different molar ratios) [5, 31, 32]. However, 3D graphitic foams, dense natural graphite, edge-rich graphene paper, and 3H3C graphene films have demonstrated less favorable cathodic performance, including low discharge capacity (~ 60–128 mAh g^−1^), insufficient cycle life with rapid capacity decay, and low rate capability due to the poor accessibility of AlCl_4_^−^ intercalation sites and poor ionic-conducting channels [24]. In addition, these materials exhibited high charge voltage plateaus in the range of 2.2–2.44 V during the charging process, only slightly lower than the battery electrolyte decomposition cutoff voltage at approximately 2.5 V. This led to severe side reactions during the charging process, resulting in inadequate discharge capacity and limited life cycles [1, 5]. Therefore, to further develop rechargeable AIBs, it is of great interest to explore new structured cathode materials that possess high energy density, exceptional long life cycle stability and lowered the charge/discharge voltage plateau near to or less than that of the electrolyte decomposition voltage.

Recently, the functional surface treatment of graphitic carbon materials (i.e., natural graphite, graphene, graphite felt) has proven to be a viable strategy for forming different pore sizes (including defect sites, large size holes, or more nanovoids) on their surface structures with a high concentration of redox-active sites without increasing their specific surface area [33–37]. Owing to the wide distribution of redox-active sites throughout the nanovoids of graphitic carbon materials, many more ions/electrons can easily penetrate/percolate into the entire material with a relatively shorter penetration depth during the charge/discharge process. Consequently, this phenomenon could contribute to an extremely high specific capacity while maintaining a favorable charge voltage plateau at nearly 2–2.2 V without any severe side reactions during the charging process. For example, Lu et al. developed plasma-etched graphene nanoribbons on highly porous 3D graphene (GNHPG) foam as a cathode material for AIBs [19]. The battery cell delivered a discharge capacity of 123 mAh g^−1^ at a rate of 5 A g^−1^ with a high discharge voltage plateau at approximately 2 V, an exceptionally long cycle life of over 10,000 cycles without any capacity decay, and superior rate performance (reversible specific capacity of 111 mAh g^−1^ even at the highest current rate of 8 A g^−1^). This superior electrochemical performance was realized through plasma etching, which induced an abundant amount of nanovoids distributed throughout the 3D graphene. The large volume of the chloroaluminate ions preferentially percolated/intercalated into the entire active material with a relatively shorter penetration depth of the AlCl_4_^−^ anions, resulting in a low charge voltage plateau at 2.3 V during the charge process. Nevertheless, this lower charge voltage plateau caused fewer side reactions leading to damaged electrolyte integrity and undesired by-products during the charge process, as well as super-stable cycling performance of GNHPG-based electrodes. In 2018, Zhang and co-workers also demonstrated edge-rich graphene paper using a low-temperature chemical vapor deposition (CVD) process [24]. The resultant edge-rich, thin graphene with abundant interconnected channels enhanced the electrolyte permeability and AlCl_4_^−^ anions/electron diffusion with a large penetration depth, which reflected a relatively high charge voltage plateau (~ 2.2 and ~ 2.43 V) during the charge process. However, a reversible capacity as high as 128 mAh g^−1^ was achieved at 2 A g^−1^ with a superior cycling stability over 20,000 cycles at a current density of 8 A g^−1^. However, the observed charge voltage plateaus were relatively higher than the electrolyte decomposition voltage, resulting in an insufficient discharge capacity in the edge-rich, graphene-paper-based cathode for AIBs.

In this study, we developed a facile process to prepare surface-modified graphitic carbon materials, namely acid-treated expanded graphite (AEG) and base-etched graphite (BEG), as cathode materials for ultra-fast charging and rechargeable AIBs. The as-prepared AEG cathode was composed of abundant micro- to nano-sized porous surface structures with an interlayer distance (*d*-spacing) of 3.371 Å and a range of different chemical environments realized by surface treatment of pristine graphite (PG) via acidic media. The AIB system incorporating AEG exhibited a specific capacity of 88 mAh g^−1^ at a current density of 4 A g^−1^ over 1000 cycles with charge voltage plateaus ranging from 2.29 to 2.35 V. The specific capacity was ~ 80 mAh g^−1^ at an ultra-high current rate of 10 A g^−1^ across 10,000 cycles with a Coulombic efficiency (CE) of approximately 99.1%. The BEG cathode consisted of abundant exposed-edge graphitic sites having different sizes of pores and large size holes or more nanovoids on the surface structure, with an expanded *d*-spacing of 3.384 Å. In addition, the oxygen-containing functionalities introduced by surface-treatment methods via KOH etching facilitated the fast, reversible chloroaluminate anions kinetics. The battery system consisting of a BEG cathode exhibited lowered charge–voltage plateaus from 2.30 to 2.35 V for 1000 cycles. In addition, BEG shows a specific capacity of 110 mAh g^−1^ at a high current density of 4 A g^−1^ with a stabilized CE near 99.7%. The battery maintains an exceptionally long life cycle of over 10,000 cycles without any capacity decay, even at an ultra-high charging rate of 10 A g^−1^ and high rate capability. Moreover, BEG also delivered a specific capacity of 98 mAh g^−1^ with a CE of 97% under ultra-fast charging at 10 A g^−1^ (about 30 s) and slow discharging at 4 A g^−1^, exhibiting a specific capacity of 100 mAh g^−1^ at a constant charging rate of 5 A g^−1^ and by varying the discharge rate from 2 to 10 A g^−1^ over 1000 cycles with a CE of 99%.

## Experimental Section

### Preparation of AEG and BEG

PG was obtained from commercial graphite (Sigma-Aldrich, < 20 μm). For the preparation of AEG, graphite powders (2 g) were treated with a mixed acidic solution (80 mL) of sulfuric acid (H_2_SO_4_, 95%, Duksan) and nitric acid (HNO_3_, 64–66%, Duksan) in a 3:1 ratio under stirring for 24 h. The reaction mixture was diluted with deionized (DI) water (200 mL). The resulting precipitate was filtered under vacuum after dilution with DI water, washed several times with DI water, and then dried at 80 °C in air overnight. The resulting mixture was finally annealed at 600 °C for 30 min in a muffle furnace to obtain the AEG specimen. Base (KOH)-etched graphite (BEG) was prepared using a solution of 4 M potassium hydroxide (KOH, 85%, Duksan). In a typical procedure, a mixture of graphite powder (2 g) and 4 M KOH solution (100 mL) was stirred vigorously at room temperature for 2 h. The resulting precipitate was filtered under vacuum and washed several times with DI water, and then the precipitate was completely dried in an oven at 80 °C. The KOH-etched graphite was annealed at 800 °C for 2 h (heating rate: 5 °C min^−1^) under N_2_ gas for the KOH activation reaction. The product was then repeatedly washed with DI water to remove the residual KOH. The final product (BEG) was collected by vacuum filtration and dried in a vacuum oven at 80 °C for 12 h.

### Material Characterization

The morphologies of the surface-treated graphite specimens were characterized by field-emission scanning electron microscopy (FE-SEM, S-4800, HITACHI) and transmission electron microscopy (FT-TEM, Tecnai G2 F20 S-TWIN, FEI) to observe the edges of the graphite and lattice directions. Information about the crystallinity, chemical bonding, and chemical structure was obtained via X-ray diffraction (XRD, DIATOME) equipped with a Cu Kα X-ray source and X-ray photoelectron spectroscopy (XPS, K-Alpha, Thermo Scientific) using an Al Kα X-ray source. In particular, the carbon material was evaluated using a micro-Raman spectrophotometer (XploRA, Horiba) using an Ar^+^ laser (532 nm). To investigate the specific surface area, pore volume, and average pore size of the graphite, nitrogen molecule adsorption and desorption was carried out using a Physisorption Ion Analyzer (BET, 3-flex, Micromeritics Instruments Corp.) at the Core Research Support Center for Natural Products and Medical Materials at Yeungnam University.

### Electrochemical Measurements

The cathode slurries were composed of surface-treated graphite (90 wt%) as the active material and PVDF (10 wt%) as a binder in *N*-methyl-2-pyrrolidone (NMP) solvent without any conducting material. The slurries were coated on a 16-mm molybdenum substrate as a current collector and dried at 120 °C for 12 h in a vacuum oven. The customized Swagelok cells were fabricated in an argon-filled glovebox to prevent oxidation of the ionic liquid electrolyte. The AIB cell was first optimized in a cell operating at 25 °C using PG, AEG, and BEG cathodes and an ionic liquid electrolyte consisting of a mixture of 1-ethyl-3-methylimidazolium chloride and aluminum chloride (AlCl_3_/[EMIm]Cl) with an optimal ratio of 1.5:1. Aluminum foil was used as the anode, and a glass microfiber filter (GF/D, Whatman) was used as the separator. To confirm the reduction/oxidation peaks, cyclic voltammetry (CV) was measured from 0.0 to 2.5 V (vs. Al/Al^3+^) at a scan rate of 0.5 mV s^−1^. The voltage range for the cycle stability and rate capability was 0.0–2.45 V (vs. Al/Al^3+^). To measure the resistance of the cells, EIS (ZIVE SP1, IVIUM Technologies) was performed from 0.1 to 10 kHz with an amplitude of 5 mV.

## Results and Discussion

### Morphological Features of AEG and BEG Cathodes

**Fig. 1:**
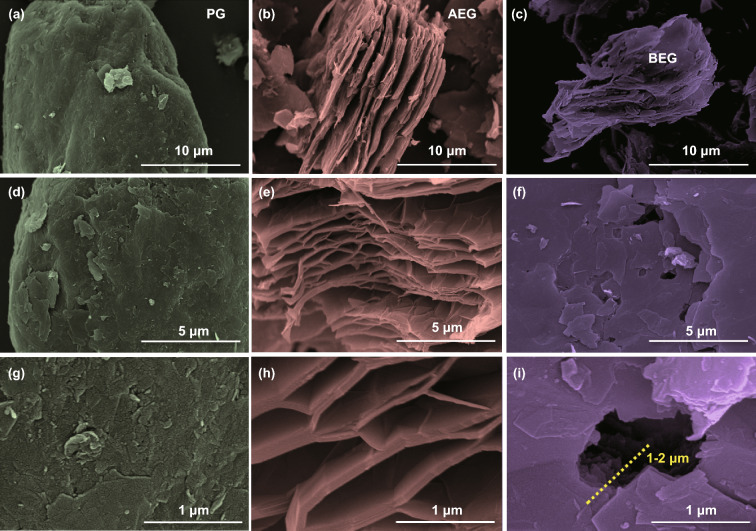
SEM images of PG, AEG, and BEG; **a–c** low resolution and **d–i** high-resolution images of samples in different magnification

Surface-treated graphite cathodes were prepared by acid-treating (AEG) and base-etching (BEG) pristine graphite (PG). In AEG, the edge of the graphitic sheets was exfoliated with abundant micron to nano-sized pores during acid treatment, which can act as redox sites for intercalation of chloroaluminate anions during the charging process. A higher volume expansion of AEG occurred (i.e., by more than threefold) compared to PG and BEG, as illustrated in the optical image in Fig. S1. The KOH etching process generates exposed-edge graphitic carbon sites with many large size holes or more nanovoids and defect sites on the surface of the BEG with a significant content of oxygen-containing functional groups, such as hydroxyl groups (C–OH), carbonyl/carboxyl groups (C=O/HO–C=O), and epoxy groups (C–O–C), which exist between the BEG layers and at the edges and defect sites. Therefore, the abundant oxygen-containing functional groups act as accessible intercalation/redox-active sites for chloroaluminate anions intercalation during the charging process and de-intercalation during the discharge process. Figure 1 displays the FE-SEM image of the PG, AEG, and BEG samples. PG particles displayed typical spherical shapes (potato-shaped graphite) with an average diameter of approximately 10–20 μm (Fig. S2), and their surface has a relatively smooth texture with irregular outer surfaces, as strongly indicated in the side and top views in Fig. 1a, d. Moreover, the smooth surface of PG was confirmed by high-resolution SEM images (Fig. 1g). In contrast, the surface morphology of AEG consists of significantly expanded graphitic layers/sheets with honeycomb-like mesoporous structures in the side view in comparison with the PG and BEG samples (Fig. 1b, e), which can be clearly seen in the high-resolution SEM images (Fig. 1h). The side view of AEG reveals widely expanded graphitic layers, yet some layers are attached, and the surface of the AEG is similar to that of PG. The BEG layers contain abundant exposed-edge graphitic carbon sites and expanded graphitic layers, as revealed in the side view of the SEM image in Fig. 1c. Moreover, the top view of the BEG exhibits several defect sites with crater morphology (i.e., large size holes or more nanovoids) on the surfaces, which were estimated to be approximately 0.5 to 1 μm in diameter and depth size about 1–2 μm (deep holes or deep craters) covered with approximately 8–10 graphite layers, as indicated by the yellow line in Fig. 1i and Fig. S3. These results indicate that many nano-sized pores are produced during the KOH treatment process. These pores are enlarged and become large size holes or more nanovoids with subsequent heat treatment at 800 °C, as obviously evidenced by the SEM images (Figs. 1i and S3). These phenomena were also observed in our previous work [37] and earlier reports by Cheng et al. [35] and Shim et al. [36] Consequently, these characteristics are more beneficial for facilitating the preferential (de)-intercalation of chloroaluminate anions into the large size holes or more nanovoids and at the edge defect sites of BEG during charge/discharge cycling. This result is further supported by the pore size distribution obtained using the Barrett–Joyner–Halenda (BJH) method (Fig. 3c) and an earlier report by Shim et al. [36].

**Fig. 2:**
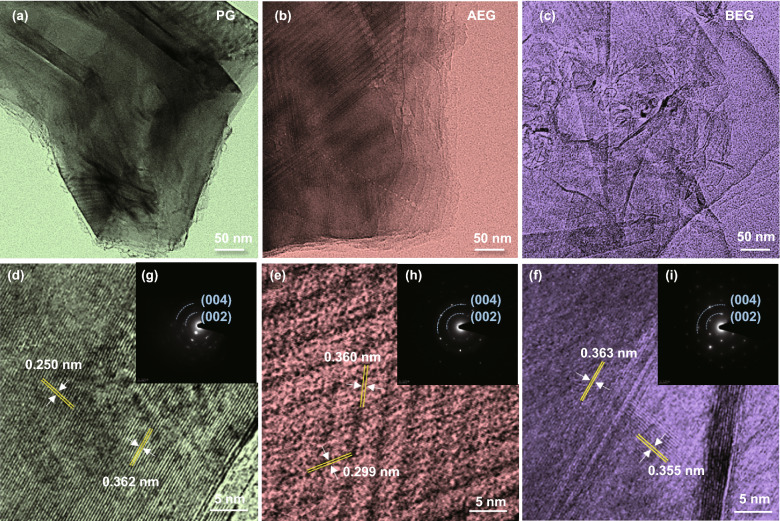
**a–c** FE-TEM images and **d–f** HRTEM images of PG, AEG, and BEG. **g–i** SAED patterns of the corresponding samples

Figure 2 illustrates TEM images of the three graphite samples. As shown in Fig. 2a, PG did not have graphitic layers at the edge, implying that the graphitic layers overlapped and aggregated. In comparison with PG, the AEG and BEG have clearly separated graphene layers/sheets at the edge of the graphite (Fig. 2b, c). Specifically, the edge of the AEG contains many graphite layers compared to that of BEG. In contrast, many large size holes with sizes of ~ 500 nm were formed, and defect nanovoids were distinctly observed on the surface of the BEG, as further evidenced in the TEM images (Fig. S4). Figure 2d‒f illustrates the high-resolution TEM (HR-TEM) images of the PG, AEG, and BEG samples with lattice fringes of the corresponding graphites. As illustrated in Fig. 2d, PG is composed of well-defined graphite lattice layers/sheets with long-range-ordered stacking approximately parallel to each other, producing well-organized turbostratic structures. The graphitic lattice directions were further elucidated by the selected area electron diffraction (SAED) pattern (Fig. 2g‒i). The PG shows two lattice directions at the (002) and (004) graphitic peaks, corresponding to interplanar spacing distances (*d*-spacing) of 0.362 and 0.250 nm, respectively. Figure 2e shows that the AEG sheets also display numerous turbostratically ordered structures with two lattice fringes (as indicated by yellow lines), indexed as the (002) and (004) planes of the graphitic sheets with *d*-spacing of 0.360 and 0.299 nm, respectively. The HR-TEM images of the sample are being formed by phase contrast, so the *d*-spacing could be larger than that calculated from diffraction pattern. Therefore, the *d*-spacing calculated from XRD (0.3371 nm) and TEM (0.360 nm) has some difference. The BEG shows many turbostratically disordered structures with two lattice directions at the (002) and (004) graphitic planes (Fig. 2f), which are associated with *d*-spacing of 0.363 and 0.355 nm, respectively. These results suggest that BEG prepared by KOH solution gave rise to turbostratic disordered graphitic structures (irregular surface distortion) with an expansion of the *d*-spacing of ~ 0.003 (002) and ~ 0.057 nm (004) in comparison with that of AEG. Consequently, as illustrated from SEM images (Figs. 1i and S3) and TEM images (Figs. 2c, f and S4) of BEG cathode, the number of graphene layers was fewer and the presence of irregular surface distortion (large size holes or more nanovoids) as compared to AEG. Hence, the presence of high density of defective sites (large size holes or nanovoids) including a significant content of redox active sites (oxygen-containing functional groups) on the surface of BEG cathode can attract and adapt more AlCl_4_^−^ ions with relatively shorter penetration depth during the charge/discharge process. Therefore, the rapid intercalation of AlCl_4_^−^ ions and successive transfer of more AlCl_4_^−^ ions on the entire surface structure as well as fewer AlCl_4_^−^ ions intercalation into the space of BEG layer has contributed to provide high BEG cathodic capacity [19].

### Characterization of AEG and BEG Cathodes

**Fig. 3:**
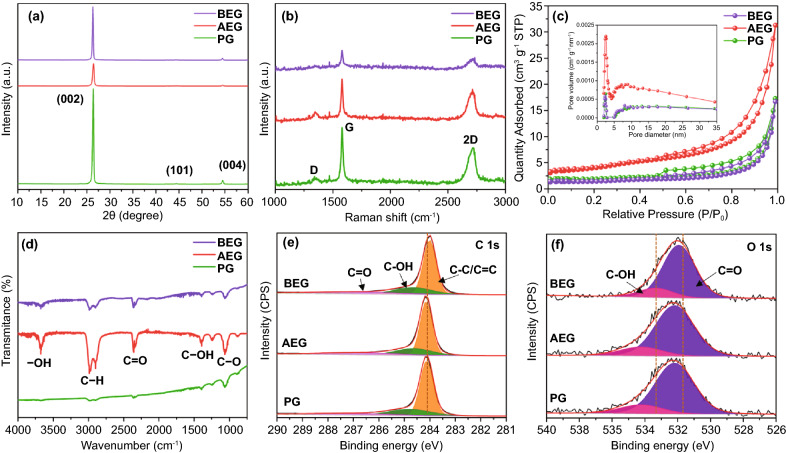
**a** XRD pattern, **b** Raman spectra, **c** BET isotherm curves (inset; BJH pore size distribution), and **d** FTIR spectra for PG, AEG, and BEG samples. The XPS profiles of **e** C 1 s signal and **f** O 1 s signal for PG, AEG, and BEG samples

Figure 3 illustrates the crystallinity, specific surface area, nature of the chemical bonds, and chemical composition of PG, AEG, and BEG samples. The XRD spectrum of PG exhibits a sharp diffraction peak at 2*θ* = 26.39° with a *d*-spacing of 3.374 Å, and the other two reflection peaks at 2*θ* = 44.4° and 54.5° correspond to the (002), (101), and (004) planes of graphite, respectively (JCPDS No. 00-008-0415) [38, 39]. As shown in Fig. 3a, the diffraction peaks diminished after surface treatment with acidic and basic media. AEG shows a decrease in the diffraction peak of the (002) plane, which is almost fivefold lower than that of PG. In addition, the intensities of the reflection peaks for the (100), (101), (103), and (110) planes also diminished after acid treatment, as seen in the XRD spectra (Fig. S5a), demonstrating that the surface treatment by the acidic solution caused the low crystallinity of the graphite particles in the AEG samples. The *d*-spacing was calculated using Bragg’s equation (*nλ* = 2*d* sin*θ*) for surface-modified graphite [40]. In AEG, the peak of the (002) plane is shifted to higher angles and located at 2*θ* = 26.42°, corresponding to a *d*-spacing of 0.3371 nm (Fig. S5b) and implying a narrowing/shrinkage in the *d*-spacing of the AEG bulk interlayers in comparison with PG (0.3374 nm). Such a shrinkage in the *d*-spacing of the AEG layers suggested the oxidation of the intercalated/interlayer groups by acid treatment, followed by the evaporation of intercalated groups as gaseous products such as CO_2_ and SO_2_ during the thermal exfoliation process at a low temperature of 600 °C. This process leads to a decrease in the *d*-spacing of AEG and a reduction in the bulk density of PG during acid treatment. In addition, volume expansion occurs with the formation of honeycomb-like microstructures, in which the open and semi-open inner pores range in size from the microscale to nanoscale (SEM images in Fig. 1b, e, h). By contrast, the diffraction peak of the (002) plane for BEG shifted to lower angles, at a position of 2*θ* = 26.31° with a *d*-spacing of 0.3384 nm, indicating an expansion in the *d-*spacing in comparison with that of PG and AEG. Such an enlarged *d*-spacing in BEG could be feasible for accelerating the reversible chloroaluminate anions intercalation kinetics. In other words, the diffusion and migration of AlCl_4_^–^ and Al_2_Cl_7_^–^ ions would become faster through a shorter penetration depth during cycles in AIBs. In addition, the calculated degree of graphitization (DG), using the equation provided in the supporting information, for BEG is much lower (65.11%) than that of both AEG (80.23%) and PG (76.74%), indicating that BEG has the turbostratic disorder structure with largely expanded *d*-spacing (*d*_002_ = 0.3384 nm) as compared to more turbostratic ordered structures of AEG (*d*_002_ = 0.3371 nm) and PG (*d*_002_ = 0.3374 nm). This feature was further well supported from HR-TEM images (Fig. 2). Therefore, BEG cathode with the turbostratically disordered structure (i.e., decreased the degree of graphitization) can be profound effect on the greater capacitive behavior, implying the intercalation of more AlCl_4_^−^ ions via the micropores, large size holes or nanovoids to improve more favorable BEG cathodic capacity comparing with AEG and PG. The crystal size *L* of each particle can be calculated by the Scherrer equation ($L = \frac{K\lambda }{{\beta \cos \theta }}$), where *K* is the constant value of shape factor (0.9–1.84), *λ* is the X-ray wavelength, *β* is the line broadening at half the maximum intensity (FWHM), and *θ* is the diffraction angle [41]. In AEG, the sizes of the crystallites comprising the (002) and (004) planes are 21.52 and 20.05 nm, respectively, which are smaller than those of PG (28.24 and 25.23 nm). It can be seen that the small crystallites of AEG (*L*_*c*_ = 21.52 nm) lead to improved electrochemical performance compared to the large crystallites of PG (*L*_*c*_ = 28.24 nm) [42]. However, the crystallite sizes of BEG (*L*_c_ = 34.74 and 28.52 nm) are much larger than those of PG and AEG. Large crystallite sizes indicate a high chloroaluminate anions intercalation capacity due to the high content of redox-active sites accessible in the large size holes or nanovoids for the electrochemical reactions. Some researchers have demonstrated that large crystallites tend to align and orient more easily than smaller crystallites, leading to higher crystallite orientation [43].

Figure 3b displays the Raman spectra of the three specimens; the PG spectrum contains three graphite peaks located at 1350, 1580, and 2715 cm^−1^. The D band located at 1350 cm^−1^ is associated with typical lattice defects at the edge of the graphitic layers. Consequently, epoxide groups are covalently bonded to the basal plane of graphite. The G band at 1580 cm^−1^ is related to the graphitic carbon structure, which implies that the C–C vibration mode of the *sp*^2^ hybrid bond is present on the surface of graphite [44]. The I_D_/I_G_ ratios for PG, AEG, and BEG were calculated to be 0.086, 0.142, and 0.097, respectively. The high I_D_/I_G_ value indicates the existence of a highly concentrated graphitic defect in the graphite sheets. As a result, AEG shows significantly higher I_D_/I_G_ values with respect to those of PG and BEG, indicating that the oxidation and thermal exfoliation process of PG introduces a high content of oxygen-containing functional groups. Such a process may cause a partial disorder with a large graphitic defect at the carbon edges of AEG [45, 46]. Raman spectrum of PG, AEG, and BEG shows very small peaks between D and G bands, which is ascribed to the defect-induced peak, indicating that PG contains defects around the edges of graphitic layers and it may result from the surface dislocations, corrugation, and carbon vacancies. Therefore, these defect sites may be suitable for promising electrochemical redox properties. In addition, the 2D band located at 2715 cm^−1^ is attributed to the second-order Raman scattering of zone-boundary phonons originating from a two-phonon double resonance, which is closely linked to the electronic band structure of graphite [44].

Figure 3c shows the Brunauer–Emmett–Teller (BET) isotherm, which provides the pore size distribution and specific surface area of the graphite particles. The shape of the nitrogen adsorption/desorption isotherm indicates the type of pore structure. The BET isotherms of the three specimens presented type H2 hysteresis, which corresponds to slit-shaped pores with non-uniform sizes, according to IUPAC [47]. Type H2 hysteresis exhibits a hysteresis loop range of P/P_0_ pressure from 0.5 to 1. The Barrett–Joyner–Halenda (BJH) shows the pore size distribution of the three specimens (inset in Fig. 3c). The pore sizes of PG and BEG exhibited large with widely distributed in the range of 2–10 nm, respectively. AEG also has a smaller pore size in the range of 2–5 nm, but a higher pore volume with respect to those of PG and BEG. The BET specific surface area of PG, AEG, and BEG was determined to be 7.85, 14.08 and 5.78 m^2^ g^−1^, respectively. Consequently, BEG has the turbostratic disorder structure with more exposed-edge graphitic carbon vacancies, fewer micropores, and large size holes or more nanovoids on the surface (defined as the high-density defective sites). This result is further well supported by BHJ test results (inset images of Fig. 3c), in which BEG showed the larger pore size diameter in the range of 2–10 nm with reduced specific surface area (BET: ca. 5.78 m^2^ g^−1^). This is probably due to the existence of large size high-density defective sites on the surface of BEG as compared to more turbostratically ordered structure of AEG (higher BET surface area: ca. 14.08 m^2^ g^−1^ and smaller pore size diameter: ca. 2–5 nm). Earlier reports have also demonstrated that the abundant holes and defect active sites of graphite plane generated by the KOH etching can provide sufficient cross-plane channels for efficient ionic diffusion, even in a highly compressed form with a low surface area. Therefore, the observed large size holes or nanovoids and defect active sites in BEG could act as excellent host sites with the electrochemical reactions, realizing the facile movement of guest chloroaluminate anions [36, 37].

The nature of the chemical bonds of the three graphite specimens was investigated by Fourier transform infrared (FTIR) spectroscopy, and the results are shown in Fig. 3d. The FTIR peaks appearing at 3600, 3000 (doublet peaks), 2350, 1390, and 1060 cm^−1^ correspond to –OH, C–H, C=O, C–OH, and C–O, respectively [48, 49]. The FTIR peaks of the AEG are more intense than those of PG and BEG, implying that the AEG has more oxygen-containing functional groups at the graphite plane or at the edge induced by the oxidizing agent. The *sp*^2^ carbon of graphite is broken by the oxidizing agent, resulting in a change in the *sp*^3^ bonds connected with many functional groups [50]. Therefore, AlCl_4_^−^ and Al_2_Cl_7_^−^ ions can react more easily with the oxygen-containing functional groups in the surface structures of AEG and BEG. The XRD and HR-TEM results reveal that the shrinkage/expansion in the *d-*spacing of the AEG and BEG samples is directly correlated to the nature and content of different carbon- and oxygen-containing functional groups in their interlayer and at the edge/defect voids. Hence, the chemical composition of carbon and the content of oxygen-containing functionalities in the graphite materials were predominantly investigated using XPS, and the typical high-resolution XPS profiles of PG, AEG, and BEG are shown in Fig. 3e, f. The C 1 s signal of three different chemically shifted components for three specimens, which could be deconvoluted into two dominant peaks at approximately 284.2 and 285 eV, is attributed to C–C/C=C bonds and C–OH bonds, respectively. A small, additional peak is located at 286.5 eV for the three specimens, which is assigned to the carbonyl groups (i.e., C=O bonds) [51]. The O 1 s signal for the three specimens is resolved into two dominant peaks centered at 531.5 and 533 eV, arising from C–OH and C=O bonds [52]. Consequently, the XPS profiles of the C 1 s and O 1 s signals for BEG show that the lower binding energy is shifted to the right compared to the spectra of PG and AEG (Fig. 3e), indicating that a greater amount of carbon- and oxygen-containing functional groups (especially for C–OH/C–O groups) were produced during the surface treatment with KOH solution. The C–OH/C–O peak for BEG was more shifted to right than that of AEG and PG, while C=O groups were almost unchanged. This result indicating the formation of abundant C–OH/C–O groups during KOH-treatment. The atomic percentages of carbon- and oxygen-containing functionalities were calculated as 97.78% and 2.22% for PG, 98.07% and 1.93% for AEG, and 97.93% and 2.07% for BEG, respectively (see XPS survey spectra in Fig. S6). It can be seen that the content of oxygen functional groups in BEG (2.07%) is lower than that of PG (2.22%), demonstrating the reduced the surface oxygen-containing groups by dissociation in the form of C–O/C–OH groups and K during KOH-treatment and annealed at 800 °C [37]. This process generates carbon vacancies on the graphite basal plane, leading to form high-density defective sites and nanopores, large size holes, or nanovoids, as illustrated in Fig. 1f, i.

### Electrochemical Performance of AEG and BEG Cathodes in AIBs

**Fig. 4:**
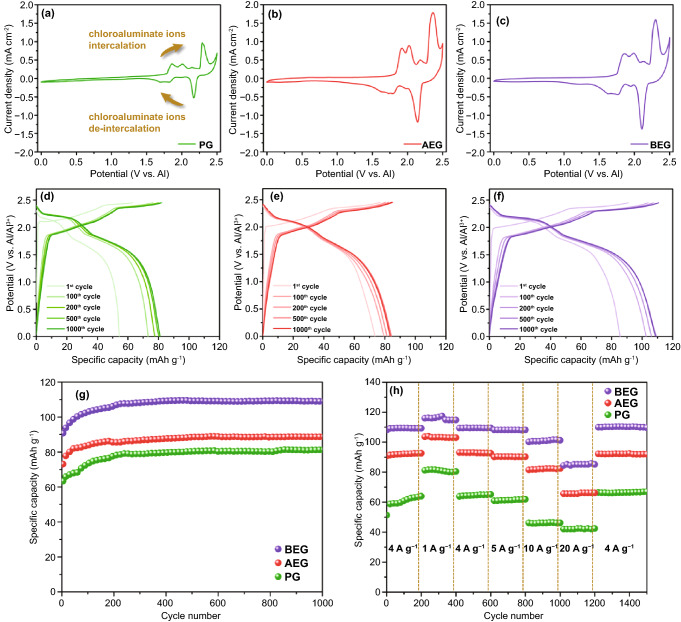
**a–c** CV curves and **d–f** charge–discharge voltage profiles at a current density of 4 A g^−1^ from 1st to 1000th cycles for PG, AEG, and BEG samples. **g** Cyclic stability of PG, AEG, and BEG samples at a current rate of 4 A g^−1^ and **h** the rate capability of PG, AEG, and BEG samples at various current densities ranging from 1 to 20 A g^−1^

CV curves were measured for each sample in the potential range of 0.0 to 2.5 V (vs. Al/Al^3+^) at a scan rate of 0.5 mV s^−1^ (Fig. 4a–c). It can be seen that AEG and BEG have well-resolved redox peaks compared with that of PG, which includes four oxidation peaks (charging/insertion) during the intercalation process and three reduction peaks (discharging/extraction) during the de-intercalation process. This demonstrates the highly reversible chloroaluminate anions (de)-intercalation kinetics in AEG and BEG [25]. The BEG voltammogram displays four distinct oxidation peaks at 1.9, 2, 2.18, and 2.35 V during the intercalation process (Fig. 4c), which are attributed to the intercalation of AlCl_4_^−^ ions into the fewer interlayer and the edge of the large size holes or nanovoids (defect sites) of BEG. The corresponding three reduction peaks are found at 1.8, 2, and 2.19 V, which are associated with the extraction of AlCl_4_^−^ ions from the fewer BEG interlayer and the edge of the defect sites. As a consequence, the simplified redox reactions of Al/graphite employing AlCl_3_/[EMIm]Cl during intercalation(charging)/de-intercalation(discharging) can be explained as follows [11, 22, 53]:

1 $${\text{Cathode:}}\quad {\text{C}}_{n} + n{\text{AlCl}}_{4}^{ - } \leftrightarrow {\text{C}}_{n} \left[ {{\text{AlCl}}_{4} } \right] + {\text{ne}}^{ - }$$

2 $${\text{Anode:}}\quad 4{\text{Al}}_{2} {\text{Cl}}_{7}^{ - } + 3{\text{e}}^{ - } \leftrightarrow {\text{Al}}^{3 + } + 7{\text{AlCl}}_{4}^{ - }$$

where *n* is the molar ratio of carbon atoms to the intercalated chloroaluminate anions in the cathodes. The balanced AlCl_4_^–^ and Al_2_Cl_7_^–^ concentrations in the electrolyte allowed for an optimal charging capacity at the cathode. There are abundant AlCl_4_^–^ ions for charging/intercalation in the cathode (Eq. 1) and a sufficient Al_2_Cl_7_^–^ concentration for discharging/electrodeposition at the anode (Eq. 2). In an AlCl_3_/[EMIm]Cl ionic liquid electrolyte, the chloroaluminate anions preferentially move through the electrolyte [1]. In addition, AEG and BEG show higher current intensity in their CV curves compared to that of PG, demonstrating a high polarization for electrochemical intercalation of AlCl_4_^−^ ions into the interlayers, at the edge or defect sites (holes or nanovoids) of AEG and BEG. These results are further supported by the diffusion-controlled processes of AEG and BEG in the electrolyte (Fig. 7). The oxidation peak located over 2.45 V is due to the electrolyte decomposition or oxidization reaction occurring on the surface of Mo [54].

To investigate the effect of redox-active site distribution on the (de)-intercalation capacity of PG, AEG, and BEG cathodes, the galvanostatic charge–discharge cycling performance in AIB cells was performed at a high current density of 4 A g^−1^, as illustrated in Fig. 4d–f. The optimized cells operating at 25 °C delivered an initial specific discharging capacity of 54.5 mAh g^−1^ for PG, 73.6 mAh g^−1^ for AEG, and 85.7 mAh g^−1^ for BEG with a CE of approximately 86.3%, 95.8%, and 94.3%, respectively. In addition, all three specimens displayed three distinct charge voltage plateaus and two discharge voltage plateaus, as evidenced by their differential capacity–voltage (dQ/dV) profiles (Fig. S7). The two major and one minor charge voltage plateaus of the three specimens are found at ~ 1.8, ~ 2, and ~ 2.35 V, and discharge voltage plateaus appeared at ~ 2.2 V and ~ 1.7, which are in good accordance with the major redox peaks found in the CV curves (Fig. 4a–c). These three charge voltage plateaus are associated with the intercalation of AlCl_4_^−^ ions into the interlayers and on the surface defect structures of PG, AEG, and BEG. The existence of interlayers, micropores, and surface defect structures would attract more AlCl_4_^−^ ions and allow to penetrate the large volume of ionic liquids to improve the cathodic capacity. Therefore, this multi-intercalation process provides the capacity rise by existing two major and one minor charge plateaus in the charge curves of PG, AEG, and BEG cathodes (Fig. 4d–f). In addition, it is noteworthy that a significant shift occurred in the discharge voltage plateaus of all three AIB cells based on PG, AEG, and BEG after initial cycles (Fig. 4d–f). In particular, PG displayed a more significant shift to higher voltage plateaus in the subsequent charge–discharge cycling compared to that of AEG and BEG, demonstrating a high polarization in the PG cathode during the insertion/extraction of chloroaluminate anions. Figure 4g shows the cycling performance of the PG, AEG, and BEG cathodes at a high current density of 4 A g^−1^. The specific capacities of PG, AEG, and BEG gradually increased from the 1st to 250th cycles. After cycling over 250 cycles, the specific capacities of PG, AEG, and BEG promptly increased to 82, 88, and 110 mAh g^−1^, respectively. Impressively, BEG exhibited the highest initial specific capacity of 85.7 mAh g^−1^ in the first cycle and retained a capacity of 110 mAh g^−1^ across 1000 cycles in comparison with PG and AEG. To investigate the rate capability, the specific capacities of the three specimens were established by applying a high charging current density from 1 to 20 A g^−1^ for 200 consecutive cycles, as shown in Fig. 4h. All three cells were first cycled at a high current density of 4 A g^−1^ for 200 cycles to obtain stable cycling states and electrode activation before the rate capability test. As a result, BEG exhibited a discharge capacity of ~ 116 mAh g^−1^ at 1 A g^−1^, ~ 110 mAh g^−1^ at 4 A g^−1^, ~ 108 mAh g^−1^ at 5 A g^−1^, and ~ 101 mAh g^−1^ at 10 A g^−1^. Even at an ultra-high current rate of 20 A g^−1^, BEG delivered the highest specific capacity of 85 mAh g^−1^, indicating a significantly better chloroaluminate anions intercalation capacity than that of PG (42 mAh g^−1^) and AEG (66 mAh g^−1^). When the current density switched rapidly from a high rate (20 A g^−1^) to a low rate (4 A g^−1^), BEG exhibited the highest reversible specific capacity of ~ 110 mAh g^−1^ compared to that of PG (66 mAh g^−1^) and AEG (92 mAh g^−1^). These results are mainly attributed to the following high-performance criteria: (i) the enlarged *d-*spacing of BEG and the turbostratically disordered structures with abundant large size holes or more nanovoids on the BEG surface for facilitating high electrolyte permeability and AlCl_4_^−^/Al_2_Cl_7_^−^ ion diffusion dynamics between the ionic electrolyte and cathode; (ii) the continuous electron/charge-conducting matrix/channels in the BEG layers through the distribution of interior active sites, which allows for fast, reversible intercalation/extraction kinetics of the chloroaluminate anions, efficient charge/current transport, and internal polarization mitigation; and (iii) the existence of the mono-layered graphene-sheet structures with a significant amount of graphitic defects at the carbon edges of BEG (I_D_/I_G_ = 0.097) compared to PG (I_D_/I_G_ = 0.086; Fig. 3b), leading to the superior electrochemical performance of BEG in fast-charging AIBs.

### Cell Performance of BEG Cathodes in AIBs

**Fig. 5:**
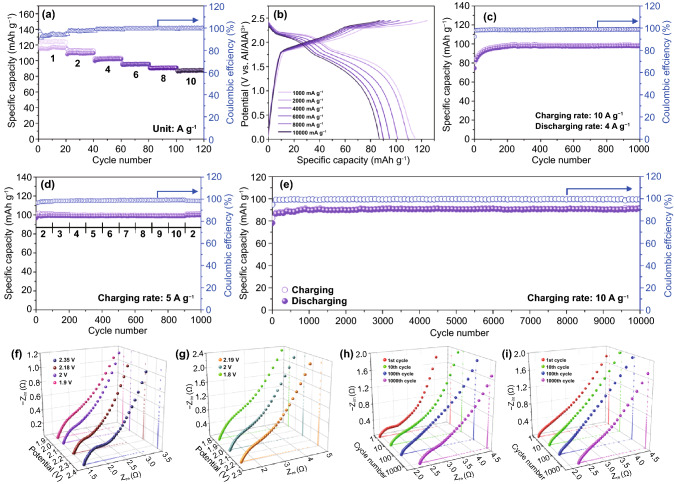
Electrochemical performance of the BEG cathode. **a** Rate performance for each current density and **b** the corresponding charge–discharge voltage profiles of **a**. **c** Cyclic stability at the fast charging rate of 10 A g^−1^ and slow discharging rate of 4 A g^−1^ over 1000 cycles, **d** Rate capability at a constant charging rate of 5 A g^−1^ with various discharging rates, and **e** Ultralong-term cyclic stability at a fast current density of 10 A g^−1^ over 10,000 cycles. Nyquist plots for various **f** oxidation potentials (intercalation) and **g** reduction potentials (de-intercalation) and cycled electrodes at **h** an oxidation potential of 2.35 V and **i** Reduction potential of 2.19 V

The diffusion coefficients of all three cathodes can be explained by the high intercalation capacity of the chloroaluminate anions. The peaks of the CV curves at different scan rates were used to calculate the diffusion coefficients of the chloroaluminate anions through the Randles–Sevick equation:

3 $$i_{p} = 268{,}600n^{3/2} AD^{1/2} Cv^{1/2}$$

where *i*_*p*_ is the maximum redox reaction peak current, *n* is the number of electrons transferred in the redox reaction (~ 1), *A* (cm^2^) is the working electrode area, *D* (cm s^−1^) is the diffusion coefficient, *C* (mol cm^–3^) is the concentration of reaction species in AlCl_3_/[EMIm]Cl, and *v* (V s^−1^) is the scan rate of CV. The diffusion coefficients (*D*_*o*_) of all redox peaks for PG, AEG, and BEG (Fig. S8), and the overall results are collated in Table S1. The BEG shows the highest *D*_*o*_ values at all redox peaks compared to those of PG and AEG. In particular, BEG exhibits *D*_*o*_ ~ 5.8 × 10^–6^ cm^2^ s^−1^ based on the maximum intensity of the oxidation peak located at 2 V, and *D*_*o*_ ~ 6.82 × 10^–6^ cm^2^ s^−1^ for the highest intensity of the reduction peak appearing at 2.2 V. In addition, the AlCl_4_^–^ diffusivities with respect to diffusion rate in PG (5.27 × 10^–6^ cm^2^ s^−1^), AEG (5.71 × 10^–6^ cm^2^ s^−1^), and BEG (5.80 × 10^–6^ cm^2^ s^−1^) are found to be approximately 2390 to 2640 times faster or greater than that of the bulk graphite and few layer of graphene films (graphitic foam), as summarized and given detailed description about the AlCl_4_^−^ ion diffusivities in Table S2. These results strongly indicate that the chloroaluminate anions are well diffused throughout the abundant large size holes, or nanovoids at the edges or in the defect sites of the BEG surface and also its wide graphitic interlayers, which results in the superior high-rate electrochemical chloroaluminate anions storage behavior and superior long life cyclic stability of BEG (Fig. 5). The electrochemical surface area (ECSA) plot of the each scan rate and the distances in the current density variation were obtained from the cyclic voltammetry curves (Fig. S9). The slope of the ECSA plot indicates the relatively higher electrochemically active surface area for BEG (0.468) than that of PG (0.448) and AEG (0.433). The plot for BEG also has an enlarged electrochemically active surface area, indicating an achievable high specific capacity. Consequently, BEG delivers an extremely high-rate electrochemical chloroaluminate anions intercalation capacity compared with PG and AEG. Therefore, only BEG has been chosen for further electrochemical tests, such as long-term cyclic stability at different high charging rates, different charge–discharge rate capability tests, AIB performance metrics at ultra-high charging rates, impedance analysis at different redox potentials, determination of capacitive/diffusion ratios, and postmortem analysis of different states of BEG electrodes using different techniques.

The electrochemical cycling stability of BEG was evaluated by charging–discharging at the highest charging rates ranging from 1 to 10 A g^−1^ over 20 consecutive cycles, as shown in Fig. 5a, b. The BEG exhibited a high specific capacity of 116 mAh g^−1^ during discharging and 124 mAh g^−1^ during charging at a current density of 1 A g^−1^ (Fig. 5a). At the highest rate of 10 A g^−1^, the specific capacity was maintained at 88 mAh g^−1^ over 20 cycles with a gradual increase in the CE to 93% and it eventually stabilized at nearly 100%. It can be seen that the values of the CE in lower of 100% observed at a current density of 1 and 2 A g^−1^, which could be attributed to the partly irreversible reactions involved in forming favorable interphases at the cathode and anode of the cell. Furthermore, the presence of excess amount of Al in the charging process induces the discharging capacity to slightly higher than that of initial capacity. Figure 5b displays the corresponding charging–discharging voltage profile, which is consistent with the result obtained in Fig. 4f. It is noteworthy that the chloroaluminate anions (de)-intercalation capacity of BEG decreases with increasing current density, which is attributed to the fact that the ultra-high charging rates can generally be influenced by the BEG cell resistance by insufficient wetting of the electrode [55]. For many practical energy applications like as LIBs, it is strategically quite meaningful for the continuous usage for a long period of time (slow discharge rate) and full charging process in a very short time (fast charge rate) in AIBs because the kinetic difference between fast charging and slow discharging could cause the deterioration or degradation of battery system performance. Therefore, the practical stability of the BEG cathode-based AIB was evaluated by the fast charging process at 10 A g^−1^ (about 30 s) and slow discharging process at 4 A g^−1^ over 1000 cycles (Fig. 5c). The process delivers the highest specific charging/discharging capacity of 98.8/97.6 mAh g^−1^ across 1000 cycles with a capacity retention of almost 100% and a CE stabilized to over 99.7%. These features suggest that the slow discharging and fast charging process could be attributed to fast chloroaluminate anions insertion/extraction kinetics in the subsequent cycles owing to wide redox-active site distribution and fewer nanopores, at the edges, and defective sites (large size holes or nanovoids) in BEG. In addition, Fig. 5d illustrates the rate performance of BEG at a constant 5 A g^−1^ charging rate and by varying the discharging rate from 2 to 10 A g^−1^; there is no distinct difference in the specific capacity upon varying the discharging rate. At the initial rate of 2 A g^−1^, the BEG delivered a CE of 98% and then eventually approached 99.9% at a high current rate of 10 A g^−1^. This result demonstrates that such a rapid charging rate and variable discharging rate could be applicable in many practical energy storage system, such as public transportation and cell phones. To evaluate the ultra-long-term cycle stability, the AIB cell based on the BEG cathode was subjected to a fast current density of 10 A g^−1^ over 10,000 cycles (Fig. 5e). It was revealed that BEG exhibited the longest life cycle (over 10,000 cycles) without any capacity fading as well as fluctuation in comparison with that of PG and AEG (Fig. S10). The BEG cathode delivered the highest specific discharge capacity of ~ 92 mAh g^−1^ after 10,000 cycles comparing with AEG (~ 80 mAh g^−1^), with an excellent capacity retention of almost 100%. This result indicates the ultra-long-term durability of the AEG and BEG cathodes cycled over 10,000 times (Figs. 5e and S10). As illustrated from SEM images (Figs. 1 and S3) and TEM images (Fig. 2), BEG has a fewer number of graphene layers and the turbostratic disordered structure due to the irregular surface distortion (i.e., high density of defective) with largely expanded *d*-spacing (*d*_002_ = 0.3384 nm), whereas AEG showed more turbostratically ordered structure (less exposed-edge graphitic carbon sites within the graphene structure) covered with abundant micro- to nano-sized pores and expanded *d*-spacing (*d*_002_ = 0.3371 nm). Consequently, the existence of these high density of defective sites (i.e., the exposed-edge graphitic carbon sites and large holes or more nanovoids) on the surface of BEG cathode can attract and adapt more AlCl_4_^−^ ions with relatively shorter penetration depth during the charge process (more capacitive-controlled process revealed from Fig. S13) compared to AEG cathode. Therefore, the rapid intercalation and successive transfer of more AlCl_4_^−^ ions on the entire surface and interlayer of BEG have contributed to provide superior charge-storage capacities with a longest life cycles in comparison with AEG cathode. By contrast, PG cathode has ~ 48 mAh g^−1^ specific capacity with unstable cycling stability from 3000 cycles to over 10,000 cycles (Fig. S10). This is probably attributed to the deterioration of PG electrode wettability and less long-standing active processes resulting from the potato-shaped structure with homogeneously expanded graphitic layers (i.e., lack of micropores and holes or nanovoids on the surface), and consequently PG showed limit the achievable loads of AlCl_4_^–^ ions during the charge process.

The superior electrochemical performance of BEG was investigated via electrochemical impedance spectroscopy (EIS) at different voltages of the chloroaluminate ions insertion/extraction process, as shown in Fig. 5f–i and its corresponding supplemented two-dimensional EIS (Figs. S11a and b) with equivalent circuit (Fig. S11c), and all impedance components are collated in Table S3. The *R*_e_ is the electrolyte (solution) resistance in the initial region on the real axis, *R*_*ct*_ is the charge transfer resistance (or polarization resistance), and *CPE*_dl_ is the constant phase element of the double layer capacitance, which is in parallel with the *R*_ct_ and Warburg impedance (*Z*_w_). The diameters of semicircles in the high- and medium-frequency regions indicate the *R*_ct_ and the *CPE*_dl_. In the low-frequency region, the *Z*_w_ is indicative of chloroaluminate ion diffusion within the electrode bulk. It is noticeable from Table S3 that all redox peaks have an identical *R*_e_ of ~ 1.5 Ω, and *R*_ct_ assumes lower values for oxidation potentials (AlCl_4_^–^ insertion; Fig. 5f) rather than the reduction potentials (AlCl_4_^–^ extraction; Fig. 5g), which indicates the preferential insertion/movement of chloroaluminate anions on the surface of the BEG electrode. Figure 5h, i shows the Nyquist plots of the 1st, 10th, 100th, and 1000th cycles for the main oxidation potential at 2.35 V and the reduction potential at 2.19 V. When the number of cycles increased from 1 to 100, the values of *R*_ct_ and *R*_e_ were less pronounced than those of the 1000th cycle (Fig. S11a, b). However, the difference in *R*_e_ at the 1000th cycle was ~ 0.27 Ω, which is negligible. Therefore, the EIS results for the ultra-long-term cyclic stability of BEG cell over 10,000 cycles indicate no dramatic change of the cell resistance during charge–discharge cycling, demonstrating the superior electrochemical performance of the BEG cathode in AIBs over PG and AEG cathodes.

**Fig. 6:**
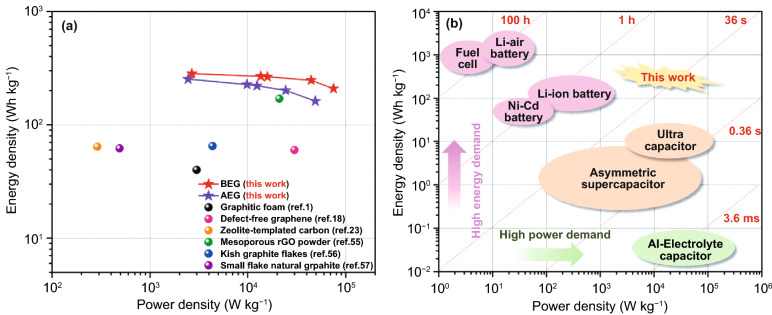
Ragone plot of energy density vs. power density for AEG and BEG cathodes: comparison with **a** state-of-the-art graphitic carbon materials reported in the literatures and **b** other energy storage systems

The energy and power density of the battery are expressed by the Ragone plot, as illustrated in Fig. 6. Based on the measured cathode capacity and current densities, the energy density and power density for BEG were calculated as 247 Wh kg^−1^ and 44.5 kW kg^−1^ at 10 A g^−1^, respectively. AEG also delivers a high energy density (201 Wh kg^−1^) and power density (24.4 kW kg^−1^). The summary plots in Fig. 6a show that BEG shows superior AIB performance metrics in terms of energy density compared with those of previously reported state-of-the-art graphitic carbon materials such as graphitic foam [1], defect-free graphene [18], zeolite-templated carbon [23], mesoporous reduced graphene oxide (rGO) powder [56], Kish graphite flakes [57], and small-flake natural graphite [53] (Table S4). Figure 6b shows the Ragone plot of the energy density vs. power density of our Al/BEG system, revealing its superior energy storage for AIBs compared to that of commercialized Li-ion batteries, supercapacitors, and electrolytic capacitors. In addition, the Al/BEG system shows a higher power density than the full cell.

### Electrochemical Investigation of the Ion Behavior at BEG Cathodes

**Fig. 7:**
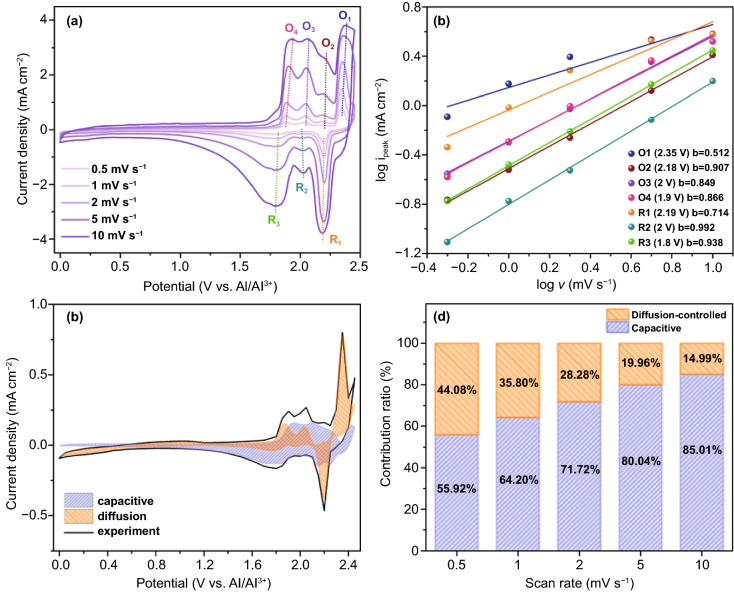
**a** CV curves for the BEG cathode at different scan rates, **b** *b*-values at different redox voltages, **c** contribution ratio of the capacitive- and diffusion-controlled process at a scan rate of 0.5 mV s^−1^, and **d** total capacity contribution ratio for the different scan rates

To better understand the excellent electrochemical performance of the BEG electrode, the charge storage mechanism and reaction kinetics were further studied according to Dunn's method [58, 59], in which CV was used. The electrochemical kinetics of the BEG electrode are governed by diffusion-controlled and capacitive-controlled processes; it is essential to know that which process is dominant. The capacitive effect is characterized by analyzing the CV current at different scan rates, as shown in Fig. 7a. The current response (*i*) is expressed by the power law:

$$i = av^{b}$$

where *i* is the current, *v* is the sweep rate, *a* is a constant, and *b* is an important indicator of the charge storage kinetics [60]. The type of contribution is indicated by the *b*-value, which determines the slope of the plot of log *v* vs. log *i* (Fig. 7b). When *b* = 1, the dominant charge storage mechanism is a surface-controlled capacitive process at the surface of the electrode. However, *b* = 0.5, suggesting that the charge storage is diffusion-controlled [61]. Figure 7b shows the *b*-values of BEG at each redox peaks, which are ~ 1, except for the peaks located at O1 (*b* = 0.512) and R1 (*b* = 0.714), indicating that the charge–discharge process in the BEG electrode has predominantly surface-controlled capacitive characteristics. The current response (*i*) at a potential (V) can be quantitatively distinguished by a two-part equation accounting for surface capacitive effects (*k*_1_ *v*) and diffusion-controlled insertion (*k*_2_ *v*^1/2^):

$$i\left( V \right) = k_{1} v + k_{2} v^{1/2}$$

is rearranged to

$$i\left( V \right)/v^{1/2} = k_{1} v^{1/2} + k_{2}$$

where *v* is the scan rate. The capacitance percentage can be determined by obtaining the values of *k*_1_ and *k*_2_. The relative contribution and the total stored charge associated with both capacitive and AlCl_4_^–^ ion insertion can be determined [62, 63]. The contribution ratio of capacitive effects was estimated at a scan rate of 0.5 mV s^−1^, as shown in Fig. 7c. The diffusion-controlled reaction region is marked by orange, which accounts for approximately 35.54% of the total stored charge. The blue region indicates the contribution of capacitive effects, which is approximately 64.46%. With increasing scan rates, the fraction of the capacitive-controlled region (blue) was higher than the diffusion-controlled region (orange), as indicated in the diffusion/capacitive-controlled contribution curves (Fig. S12). The total contribution ratios of the capacitive- and diffusion-controlled processes in the EPG electrode at different scan rates are shown in Fig. 7d. It is noted that the percentage of the capacitive region gradually increases as the scan rate is increased from 0.5 to 10 mV s^−1^. The ratios of 55.92% and 85.01% mainly arose from the capacitive-controlled process at 0.5 and 10 mV s^−1^, respectively. These results strongly indicate that the superior rate performance of BEG for AIBs originates from the high contribution ratio of the capacitive-controlled process. This indicates a dominant capacitive behavior during the charge–discharge process of the BEG electrode while maintaining the fast redox reaction kinetics and rate-independent behavior during cycling. With the increase in scan rates, the fraction of the diffusion-controlled process decreased due to insufficient time for the AlCl_4_^–^ ions to preferentially insert into the interior active sites of the BEG electrode [64]. In addition, the total contribution ratios of the capacitive- and diffusion-controlled processes of PG, AEG, and BEG revealed that the capacitive character (blue region) increased as the scan rate increased, resulting in a linear relationship (Fig. S13). The slope indicates that the surface-modified graphites have greater electrochemical reaction sites for efficient chloroaluminate ions storage. The AEG had the highest slope value of 4.569 compared to PG (4.521) and BEG (3.701), indicating the highest amount of reaction sites. Nevertheless, BEG has a larger capacitive region owing to many large size holes or more nanovoids and defect sites on the surface of the BEG graphitic layers. The possible reason is the fact that the electrochemical quantities (i.e., electron transfer rate, capacitance and density of electronic states) are mainly dependent on the defect density of the basal plane of graphite. As illustrated in SEM images (Figs. 1 and S3) and TEM images (Figs. 2 and S4), BEG comprises the high-density defective sites on the surface structure with large size holes or more nanovoids as compared to AEG. That is why BEG showed larger pore size diameter in the range of 2–10 nm with significantly reduced estimated surface area (BET: ~ 5.78 m^2^ g^−1^) as compared to those of AEG (higher BET surface area: ~ 14.08 m^2^ g^−1^ and reduced pore size diameter: 2–5 nm). As a result, these large size holes or more nanovoids on the surface/defect edge of BEG would attract and adapt more AlCl_4_^−^ ions through the penetration of large volume of ionic liquids to produce robust pseudocapacitive charge-storage amount compared to with PG and AEG (Fig. S13). Therefore, BEG exhibited a high portion of capacitance effect in the whole capacity in comparison with that of AEG and PG.

**Fig. 8:**
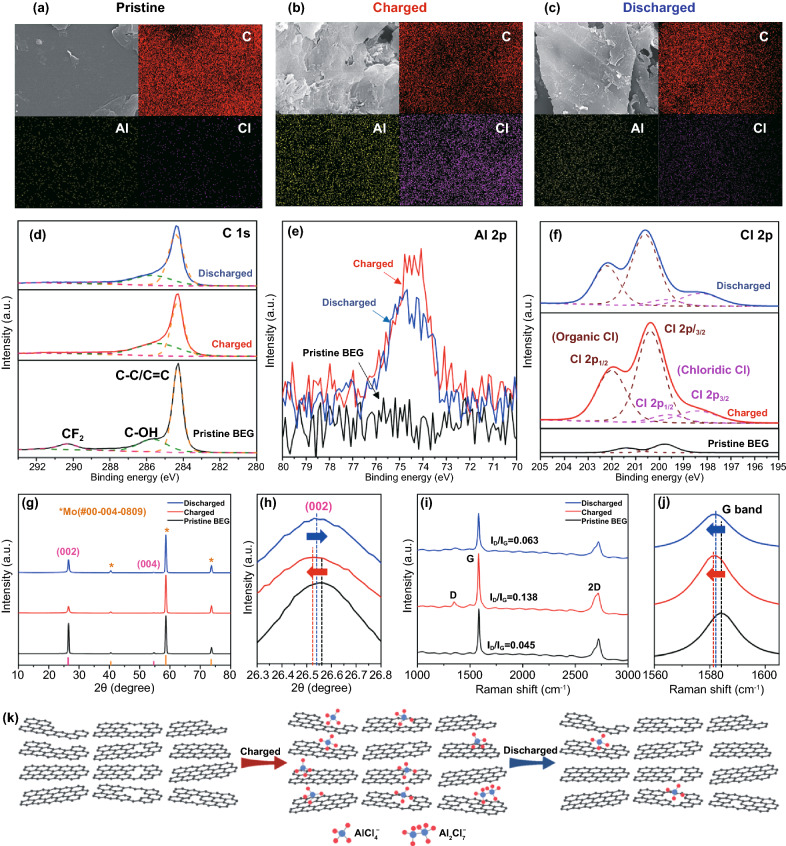
SEM–EDX analysis of **a** pristine, **b** charged, and **c** discharged BEG electrode. The XPS profiles of **d** C 1s, **e** Al 2p, and **f** Cl 2p signals, **g** XRD patterns, **h** the enlarged spectrum of (002) plane peaks for different states of BEG electrode. **i** Raman spectrum for different states of the pristine, charged, and discharged BEG electrode, and **j** the enlarged spectrum of the G band peaks for comparison. **k** Schematic diagram of the transportation of chloroaluminate anions into BEG electrode during charge–discharge cycling

To compare the microstructures of the BEG surface before and after charged/discharged cycled electrodes, SEM–energy-dispersive X-ray (SEM–EDX) mapping of the BEG electrodes over 1000 cycles is shown in Fig. 8a–c. Before cycling, the pristine electrode of the BEG microstructure has a smooth surface with a high concentration of carbon and a lower content of Al and Cl (Fig. 8a). However, the surfaces of the electrodes have seemingly blurred films after cycling, as evident in the SEM images in Fig. 8b, c, which is mainly attributed to the decomposition of the electrolyte and side reactions occurring during the charging and discharging processes. The SEM–EDX images clearly reveal strong Al and Cl element signals uniformly distributed over the entire BEG electrode surface at a charged state of 2.45 V. Furthermore, the distribution of these element signals (Al and Cl) was markedly weaker in the discharged state (0.0 V). These results provide strong evidence that AlCl_4_^−^ is preferentially intercalated into the defective sites (i.e., large size holes or nanovoids) of the BEG graphitic layers during the charging process; this conclusion is supported by the blurrier film surface in the charged state and strong Al and Cl signals in Fig. 8b [64]. Moreover, the remaining Al signals in the fully discharged state are tentatively attributed to the irreversible intercalation of Al species (AlCl_4_^−^ and Al_2_Cl_7_^−^) into the graphitic layers at the edges or defect sites (Fig. 8c). Weak Cl signals were also observed in the discharged state, which is ascribed to the irreversible side reactions between *sp*^2^ carbon and Cl^−^ species at high voltages [65]. Ex-situ XPS studies were carried out to reveal the chemical state of the BEG electrodes at charging/discharging states. Figure 8d–f shows the XPS profiles of C 1s, Al 2p, and Cl 2p signals for the pristine BEG electrode, BEG electrode fully charged to 2.45 V, and BEG electrode discharged to 0.0 V, respectively. The XPS profile of C 1s signals displays two dominant peaks at 284.3 and 286 eV for the pristine, charged, and discharged states of the BEG electrode (Fig. 8d), which correspond to the binding energies of C–C/C=C bonds and C–OH groups, respectively. The small peak appearing at 290 eV can be assigned to the CF_2_ groups of the PVDF binder in BEG electrode [66]. When the electrodes were fully charged to 2.45 V and discharged to 0.0 V, the intensities of the dominant C–C/C=C and C–OH peaks are markedly diminished, demonstrating the reduction of redox-active sites and C–OH groups as a result of the intercalation of AlCl_4_^−^ into the multichannel, open defects, holes or nanovoids structure of BEG. Figure 8e, f illustrates that the Al 2p peak (74.5 eV) and Cl 2p peak (200.5 eV) become more pronounced in the fully charged state (2.45 V) relative to that of the fully discharged state (0.0 V). These peaks are relatively weak for the pristine BEG electrode. The XPS profile of the Cl 2p signal shows a decrease in the intensity of the organic Cl 2p_1/2_ peak (202.1 eV) after discharging to 0.0 V, demonstrating the formation of C–Cl bonds as a result of de-intercalation of AlCl_4_^−^ ions. These results reveal the intercalation of AlCl_4_^−^ and Al_2_Cl_7_^−^ during the charging process [67, 68]. Moreover, a substantial reduction of the Al and Cl element signals was apparent in the fully discharged state, indicating extraction of AlCl_4_^−^ ions. Ex situ XRD was also used to monitor the crystal structure changes in the different states of the BEG electrode, as shown in Fig. 8g, h. When the charged state is 2.45 V, the (002) plane peak intensity is decreased compared to that of the pristine BEG. This could be attributed to the intercalation of AlCl_4_^−^ ions into the large size holes or nanovoids and at the edge/defect sites of the BEG layers during the charging process. As the BEG electrode was fully discharged to 0.0 V, the (002) plane peak increased in intensity compared with the charged state, demonstrating the extraction of AlCl_4_^−^ ions from the defective sites of BEG layers. In addition, the (002) plane peak is significantly shifted to lower 2*θ* values (2*θ* = 26.52°, *d*_002_ ~ 3.367 Å) in the charged state (2.45 V) with respect to that of the pristine (2*θ* = 26.56°, *d*_002_ ~ 3.362 Å) and discharged state (2*θ* = 26.55°, *d*_002_ ~ 3.363 Å). This result strongly suggests that the energy-storage mechanism of BEG involves the incorporation of more AlCl_4_^−^ ions into the exposed graphitic carbon sites and large size holes or nanovoids of the BEG layers [67]. This intercalation mechanism of AlCl_4_^−^ was further confirmed by ex situ Raman spectroscopy of the BEG electrodes in the charge and discharge states, as shown in Fig. 8i, j. The Raman spectra exhibited two main peaks at 1350 and 1592 cm^−1^, corresponding to the D and G bands, respectively. These peaks become more intense and shift to lower Raman frequency (1591.5 cm^−1^) when charged to 2.45 V relative to those of the discharged state and pristine BEG electrodes. The I_D_/I_G_ ratios for the pristine, charged, and discharged states of the BEG electrode were calculated as 0.045, 0.138, and 0.063, respectively (Fig. 8i). These results strongly indicate that the AlCl_4_^−^ ions mainly participate in the energy storage capacity during the charging process. The 2D band also became broader with significantly increased intensity at 2.45 V in comparison with that of the discharged and pristine electrodes. Moreover, the G band peak for the charged and discharged states is shifted to lower frequencies (1581.7 and 1581.9 cm^−1^, respectively) relative to the pristine state (1584.1 cm^−1^), as indicated by the arrow in Fig. 8j. Figure 8k is the schematic diagram of insertion/extraction of AlCl_4_^−^ into the BEG graphitic layers. In charging process, chloroaluminate anions (AlCl_4_^−^ and Al_2_Cl_7_^−^ ions) insert to the graphitic sheets through large size holes or nanovoids, the edge and defect sites, which can have fast insertion kinetics. Furthermore, when AlCl_4_^−^ ions extract from the BEG layers (discharging process), which allows for fast, reversible extraction kinetics and efficient charge transport. A comparison table is provided to highlight our results (Table S5), which show that the surface-treated graphite cathodes (AEG and BEG) have superior AIB performance metrics in terms of high specific capacity at ultra-high charging rates; a super-stable, long life of up to 10,000 cycles; and a CE stabilized to nearly 100% compared to that of previously reported state-of-the-art graphitic carbon materials. We also summarized the introduction of different technologies that could generate adequate different surface defects on the graphitic carbon materials and their effect on the (de)intercalation capacities, as shown in Table S6. Among the different methods that process different high density of surface defects, such as vacancy holes and polygons, deep craters, large size defects, 3D mesh network and nanovoids, high volume of pore regularity, and fragmentized particles, our methods of acid- and base-etched treatment (i.e., AEG and BEG) have been shown to exhibit profound effect on the attract and uptake of more AlCl4 − ions through the penetration of large volume ionic liquids into surface defects rather than only surface/space modified cathodic materials, finally leading to the superior performance of AIBs at an ultra-fast charging current rates. Therefore, this findings revealed that enlarging the graphite *d*-spacing, the generating exposed graphitic carbon sites, large size holes or more nanovoids at the edge/defect sites in the graphitic plane are more beneficial to attain efficient diffusion dynamics and reversible (de)-intercalation kinetics of chloroaluminate anions towards extremely high storage capacity and stable performance in AIBs.

## Conclusions

In summary, we developed ultra-fast charging AIBs using surface-treated graphitic cathode materials with superior performance. The highest specific capacities were achieved about 89 and 110 mAh g^−1^ at 4 Ag^−1^ for the AEG and BEG cathodes, respectively. Both AEG and BEG cathodes showed a super-stable cycling life of 10,000 charge–discharge cycles without any capacity decay even at ultra-high current densities, along with a high discharge voltage plateau near 2.2 V. The Al/BEG cells exhibited an ultra-high rate performance in comparison with that of the Al/AEG and Al/PG cells owing to the wide distribution of redox-active sites at the edge and over the surface defects (i.e., large size holes or nanovoids) of the BEG cathode introduced by the KOH etching process. At the same time, the expanded graphitic interlayers were maintained in BEG, which accelerated the diffusion dynamics and efficient (de)-intercalation kinetics of AlCl_4_^−^ storage even at fast charging current rates. Our fabricated Al/AEG and Al/BEG battery cells can afford energy densities of ~ 201 and ~ 247 Wh kg^−1^ at high power densities of up to ~ 49.1 and ~ 75.1 kW kg^−1^, respectively, which are higher than those of lithium-ion batteries, supercapacitors, and electrolytic capacitors, as detailed in the Ragone plot. Therefore, our findings contribute to the development of surface-modified graphitic carbon materials as cost-effective, safe, and fast-charging advanced electrode materials for high-energy–density AIBs.

## Supplementary Information

Below is the link to the electronic supplementary material.

Supplementary file1 (DOCX 4803 kb)
