# Supplementary material for: High-Defect-Density Graphite for Superior-Performance Aluminum-Ion Batteries with Ultra-Fast Charging and Stable Long Life
Source: Nanomicro Lett. 2021 Aug 9;13:171. doi: 10.1007/s40820-021-00698-0 (PMC8353050; doi:10.1007/s40820-021-00698-0)
Supplement: Supplementary file 1 — Supplementary file1 (DOCX 4803 kb) [file 40820_2021_698_MOESM1_ESM.docx]

Supporting Information for

**High-Defect-Density Graphite for Superior-Performance Aluminum-Ion Batteries with Ultra-Fast Charging and Stable Long Life**

Jisu Kim^1^, Michael Ruby Raj^1^, and Gibaek Lee^1,^*

^1^Advanced Energy Materials Design Lab, School of Chemical Engineering, Yeungnam University, 38541 Gyeongsan, Republic of Korea

*Corresponding author.

E-mail: gibaek@ynu.ac.kr (Gibaek Lee)

S1. Experimental Section

**Calculation method for the degree of graphitization.**

The degree of graphitization (DG) of graphite can be defined as the level of the transformation of non-graphitic carbon material into a well-ordered graphitic structure. DG can be calculated from the following equation [S1]:

DG (%) = $\frac{0.3440-d(002)}{0.3440-0.3354} \times$ 100 (1)

where 0.3440 nm is the *d*-spacing of the fully non-graphitized carbon (nm), 0.3354 nm is the *d*-spacing of the ideal graphite crystallite (nm), and *d*_(002)_ is the *d*-spacing derived from the XRD pattern of the studied materials (PG, AEG and BEG).

**Calculation methods of energy density and power density.**

We have calculated using modified equation as per earlier report [S2-S4]. Specific energy density (*E_sp_*) and power density (*P_sp_*) of the BEG was calculated using following equation.

*E_sp_* = $\frac{V\times I\times t}{m}$ *(Wh kg^-1^)* (2)

*P_sp_* = $\frac{V\times I}{m}$ *(W kg^-1^)* (3)

Where *V* is the operating voltage, *I* is the current (A), *t* is the time of the discharge cycle (h), *m* is the loading mass of cathode active material (kg).

S2. Supplementary Figures and Tables


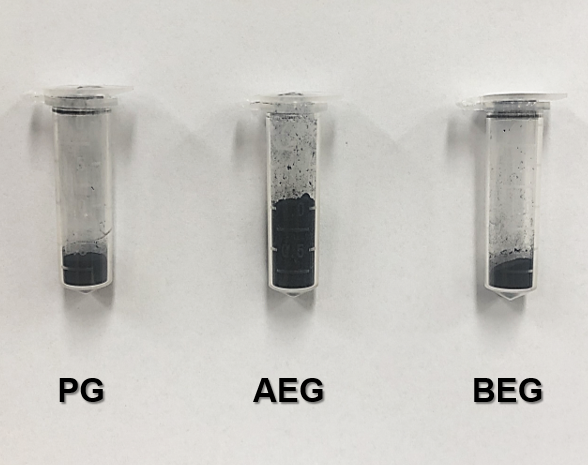


**Fig. S1** Optical images of 0.1 g samples of PG, AEG, and BEG.

**
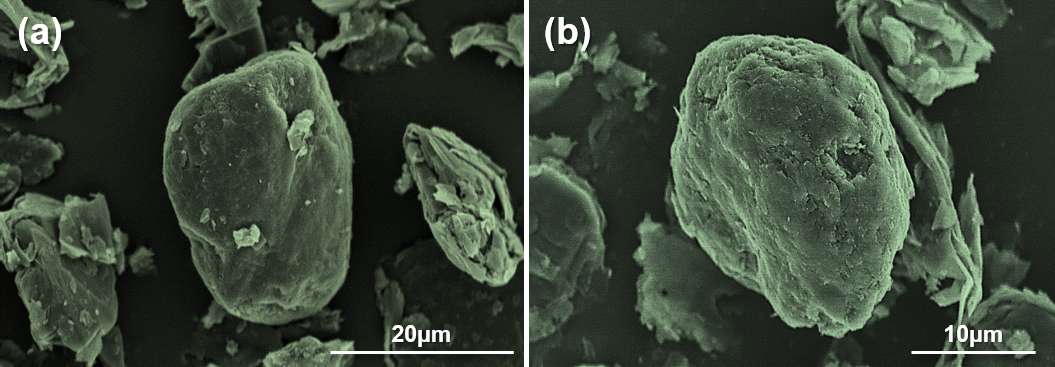
**

**Fig. S2** **a** and **b** Low-resolution SEM images for potato-shaped PG specimen.

**
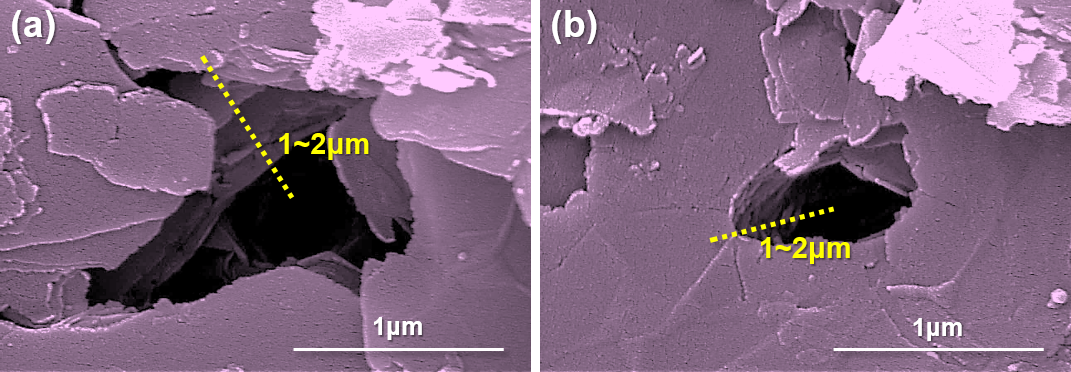
**

**Fig. S3 a** and **b** SEM images of the KOH-etched graphite (BEG). BEG surface comprise large size of 1~2 μm deep holes with approximately 8~10 graphite layers, which could facilitate the penetration of large volume of ionic liquid and more AlCl_4_^−^ ions within the BEG rather than only surface of BEG.

**
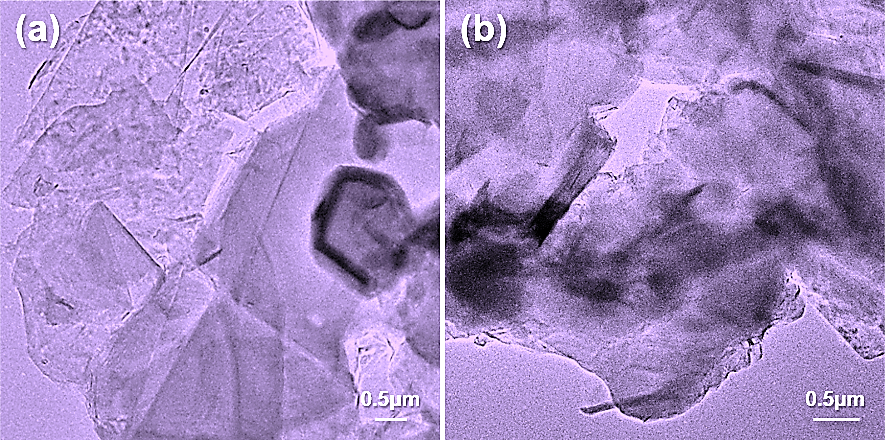
**

**Fig. S4** **a** and **b** TEM images of BEG. The pores/holes are about 500 nm in size.


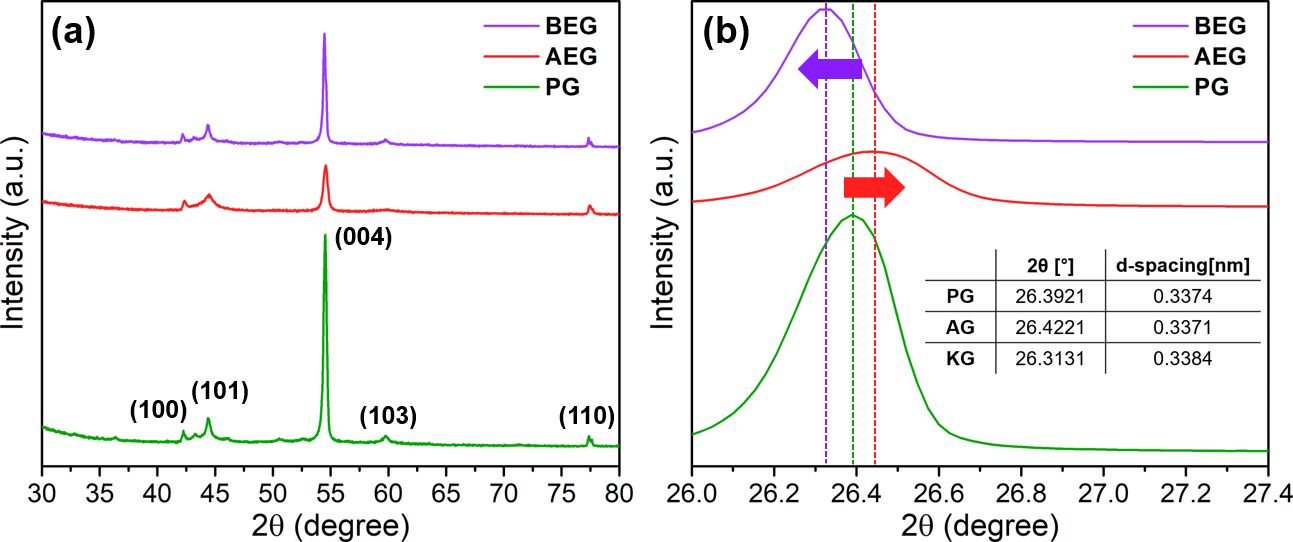


**Fig. S5** XRD pattern **a** from 30–80° and **b** the enlarged spectrum for the (002) plane peak of three specimens.

**
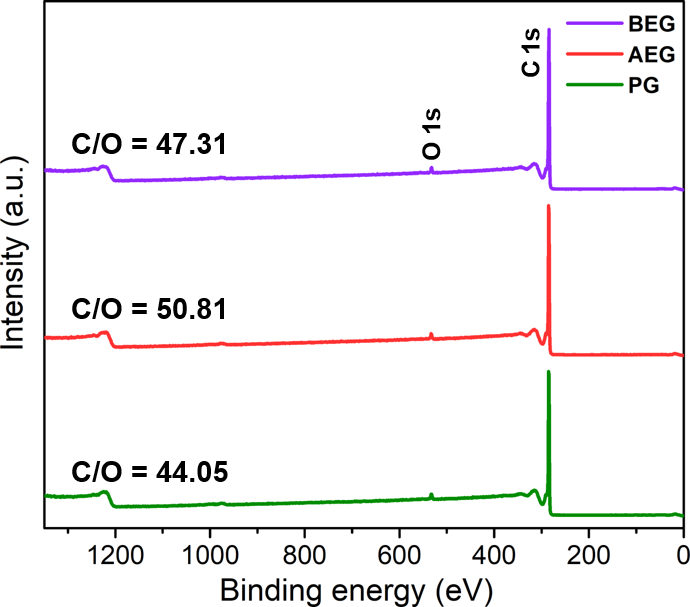
**

**Fig. S6** XPS survey spectra of PG, AEG and BEG with ratio of C and O elements.

**
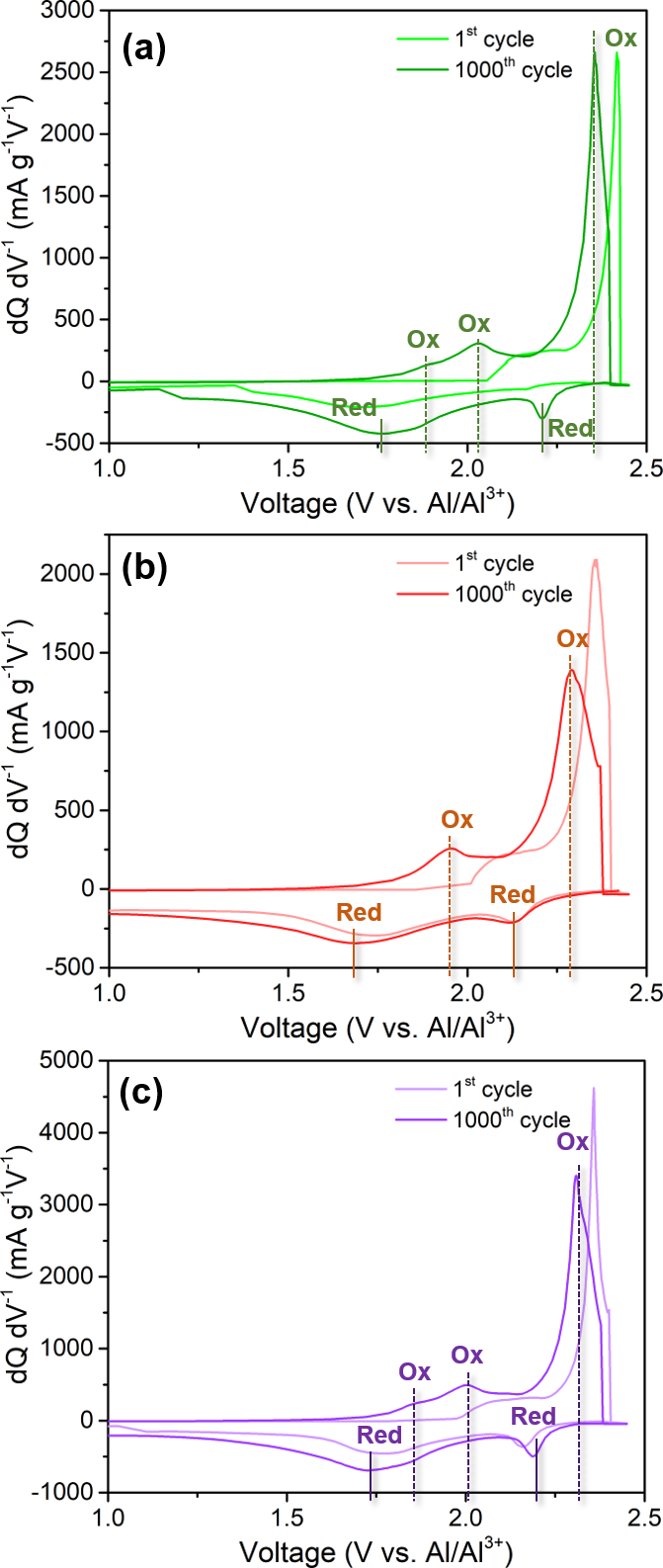
**

**Fig. S7** Differential capacity–voltage (dQ/dV) profile for **a** PG, **b** AEG, and **c** BEG.


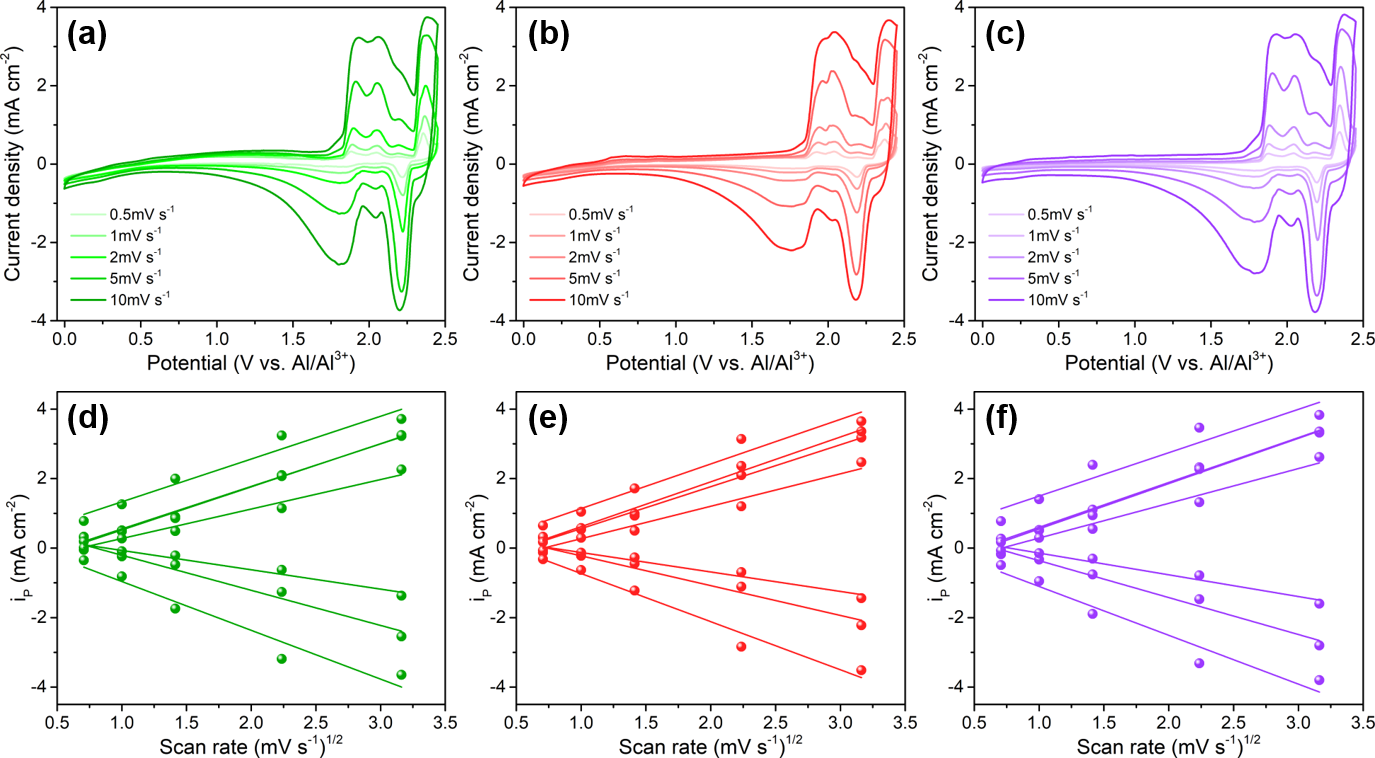


**Fig. S8** CV curves at different scan rates of **a** PG, **b** AEG, and **c** BEG. **d–f** Relationship between square root of scan rate (*v*^1/2^) and peak current (*i*_p_) of each specimens.

**
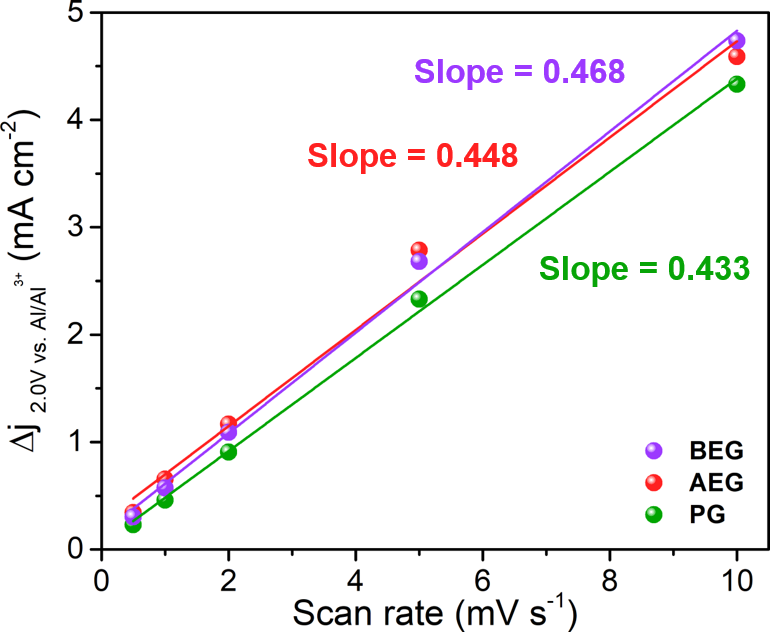
**

**Fig. S9** Relationship between scan rate and distances in current density variation (∆*j* = *j*_a_ -*j*_c_) at a potential of 2.0 V. The linear slope is the double layer capacitance (*C*_dl_) of the specimens and can be used to calculate the relative electrochemically active surface area.


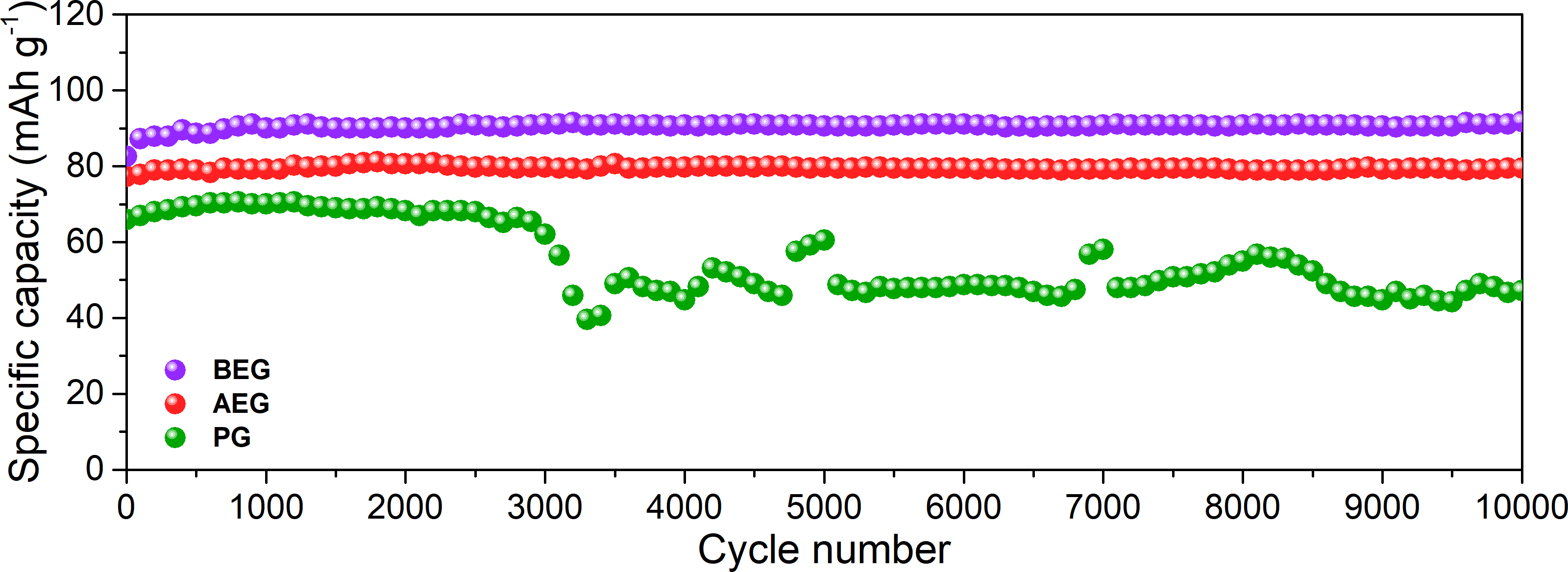


**Fig. S10** Ultralong-term cyclic stability of PG, AEG and BEG at an ultra-high current density of 10 A g^–1^ over 10000 cycles.


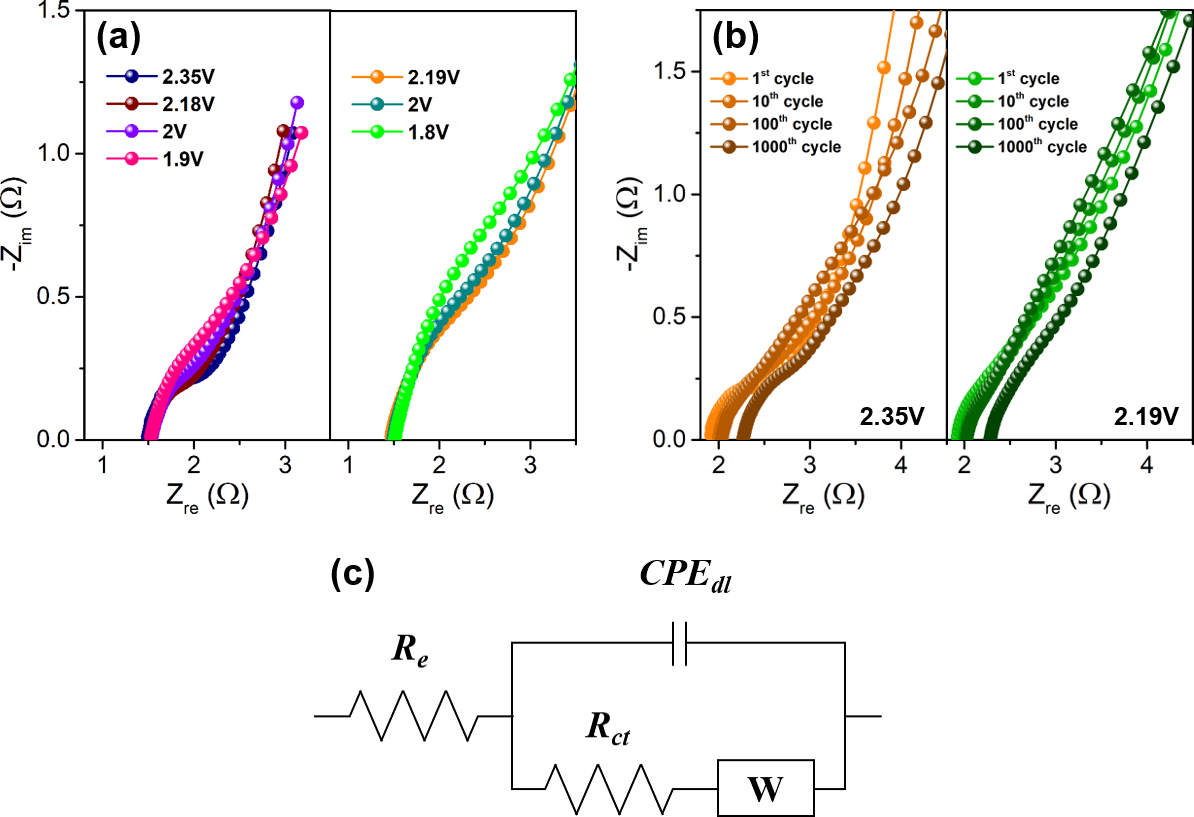


**Fig. S11** Two-dimensional Nyquist plot of **a** various oxidation potentials (intercalation) and reduction potentials (de-intercalation); **b** 1^st^ cycle →1000 cycled BEG at oxidation potential of 2.35 V and reduction potential of 2.19 V. **c** Equivalent circuit corresponding **a** and **b**.

**
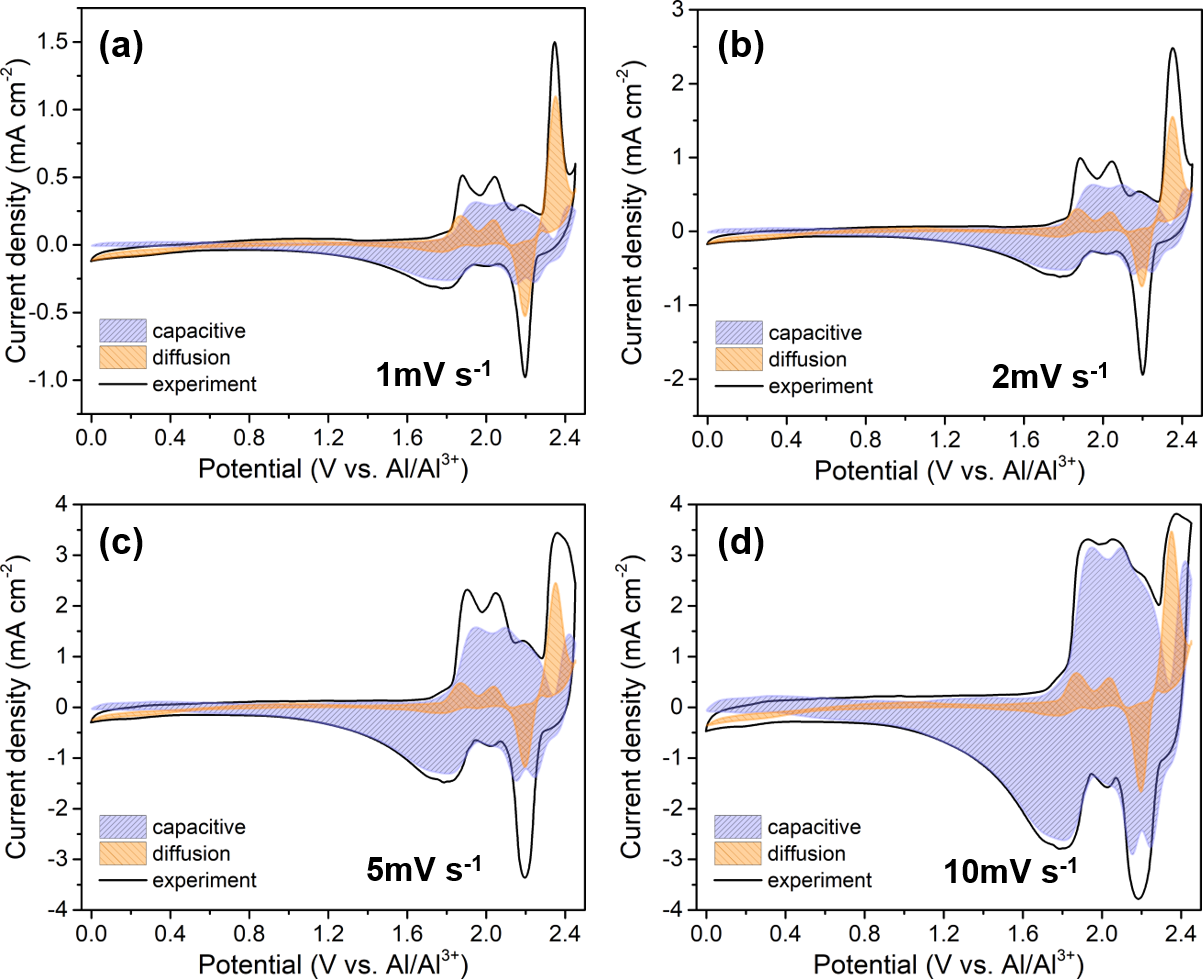
**

**Fig. S12** Capacitive- and diffusion-controlled contribution curves of BEG at a scan rate of **a** 1 mV s^–1^, **b** 2 mV s^–1^, **c** 5 mV s^–1^, and **d** 10 mV s^–1^.

**
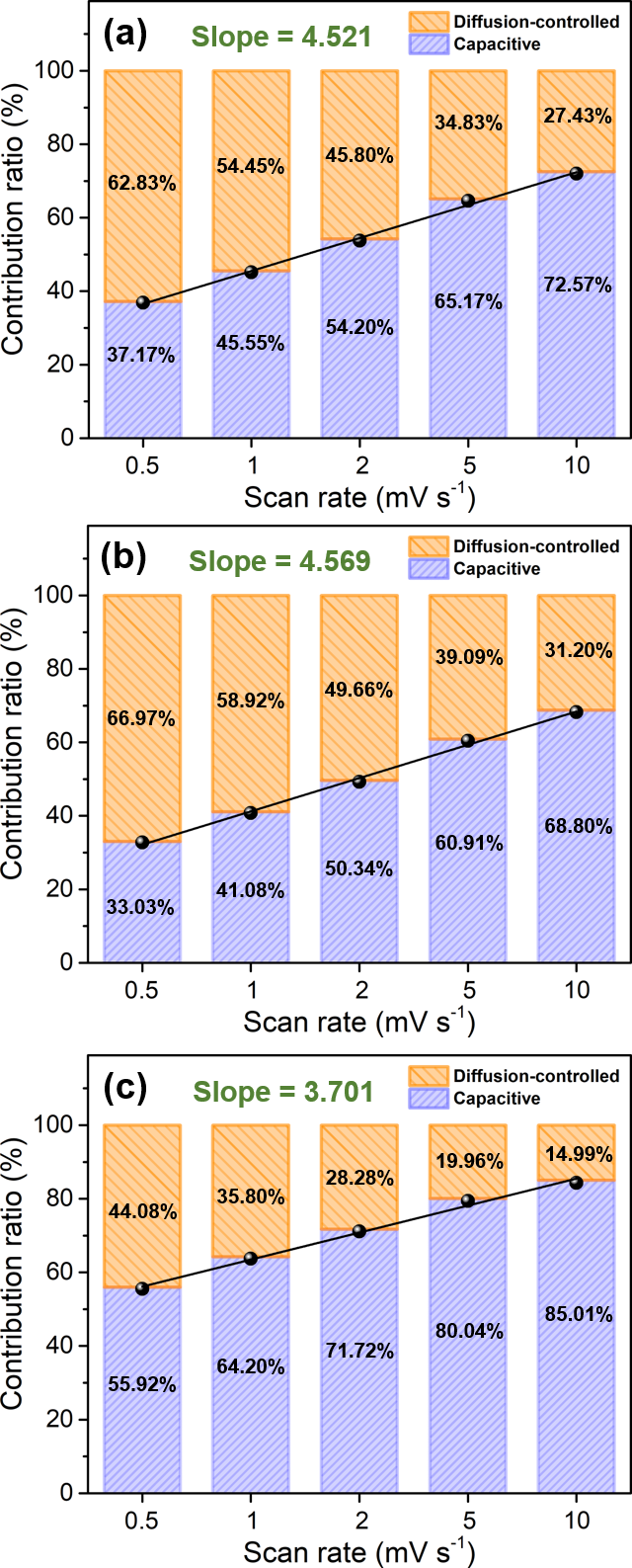
**

**Fig. S13** Total contribution ratios of capacitive and diffusion: **a** PG, **b** AEG, and **c** BEG.

**Table S1** Summary of diffusion coefficient (*D_o_*) values for different redox peaks for PG, AEG, and BEG specimens.

| $\times$10^-6^ | **Oxidation potentials** | | | | **Reduction potentials** | | |
| --- | --- | --- | --- | --- | --- | --- | --- |
|  | **1.9 V** | **2.0 V** | **2.2 V** | **2.35 V** | **1.8 V** | **2.0 V** | **2.2 V** |
| **PG** | 5.10 | 5.27 | 2.42 | 5.19 | 3.50 | 1.04 | 6.72 |
| **AEG** | 5.02 | 5.71 | 2.97 | 5.62 | 2.54 | 1.08 | 6.53 |
| **BEG** | 5.71 | 5.80 | 3.43 | 5.36 | 3.85 | 1.36 | 6.82 |

**Table S2** Comparison for the AlCl_4_^–^ Diffusivities (*D)* of our materials (PG, AEG and BEG) with bulk graphite and few-layer graphene films.

| **System** | ***N*** | ***D* (cm^2^ s^–1^)** | ***D/D_graphite_*** | **Ref** |
| --- | --- | --- | --- | --- |
| **Bulk graphite** | ‒ | 2.2 × 10^–9^ | 1.0 | [S5] |
| **Few-Layer Graphene Films (graphitic foam)** | 6 | 3.3 × 10^–9^ | 1.5 | [S5] |
|  | 5 | 2.3 × 10^–8^ | 10.2 |  |
|  | 4 | 1.1 × 10^–7^ | 47.8 |  |
|  | 3 | 3.4 × 10^–7^ | 153.2 |  |
|  | 2 | 5.0×10^–7^ | 225.2 |  |
| **PG** | ‒ | 5.27×10^–6^ | 2395.4 | This work |
| **AEG** | ‒ | 5.71×10^–6^ | 2595.4 |  |
| **BEG** | ‒ | 5.80×10^–6^ | 2636.3 |  |

**N* represents the number of layers in graphene film.

**Table S2** summarizes the absolute and relative AlCl_4_^−^ ion diffusivities with respect to bulk graphite and few-layer of graphene films (graphitic foam). It can been seen that AlCl_4_^–^ diffusivity increased markedly as the number of graphene layers decreased in graphitic foam [S5]. The diffusivity in the five-layer graphene film (2.3 × 10^–8^ cm^2^ s^–1^) is 10.2 times faster than that in bulk graphite (2.2 × 10^–9^ cm^2^ s^–1^), indicating that AlCl_4_^–^ ion diffusivity increased in graphitic foam from five to two graphene layers. The following trend, the AlCl_4_^–^ diffusivities in PG (5.27×10^–6^ cm^2^ s^–1^), AEG (5.71×10^–6^ cm^2^ s^–1^) and BEG (5.80×10^–6^ cm^2^ s^–1^) are approximately 2390 to 2640 times faster than that of the bulk graphite. This result manifesting the high AlCl_4_^–^ diffusivities (ionic conductivity) of PG, AEG and BEG compared to that of bulk graphite [S5]. Hence, it is interpreted that the experimentally observed the diffusion coefficient (AlCl_4_^–^ diffusivities) with respect to diffusion rate in PG, AEG and BEG cathode are greater than that of bulk graphite and graphitic foam.

**Table S3** Summary of EIS results for BEG cathode at all redox peaks before cycling.

| **[Ω]** | **Oxidation potentials** | | | | **Reduction potentials** | | |
| --- | --- | --- | --- | --- | --- | --- | --- |
|  | 1.9 V | 2.0 V | 2.18 V | 2.35 V | 1.8 V | 2.0 V | 2.19 V |
| ***R*_e_** | 1.53 | 1.53 | 1.53 | 1.49 | 1.51 | 1.49 | 1.47 |
| ***R*_ct_** | 1.42 | 1.07 | 1.02 | 0.91 | 3.49 | 2.53 | 2.50 |

**Table S4** Comparison of power density and energy density for AEG and BEG electrodes with other graphitic carbon materials.

| Cathode materials | Power density  [W kg^-1^] | Energy density  [Wh kg^-1^] | Refs. |
| --- | --- | --- | --- |
| Graphitic foam | ~3,000 | ~40 | [S6] |
| Mesoporous rGO powder | 21,000 | 170 | [S7] |
| Kish graphite flakes | 4,363 | 65 | [S8] |
| Defect-free graphene | 30,000 | 60 | [S9] |
| Small flake natural graphite | 489 | 62 | [S10] |
| Zeolite-template carbon | 290 | 64 | [S11] |
| Graphite | ‒ | 135 | [S19] |
| Vein graphite | ‒ | 59.1 | [S21] |
| Base-etched graphite  (BEG) | **2,680** | **282.98** | **This work** |
|  | **13,583** | **268.03** |  |
|  | **15,903** | **265.04** |  |
|  | **44,497** | **247.21** |  |
|  | **75,058** | **208.50** |  |
| Acid-treated expanded graphite (AEG) | **2,453** | **252.84** |  |
|  | **9,830** | **226.63** |  |
|  | **12,431** | **220.99** |  |
|  | **24,434** | **200.90** |  |
|  | **49,124** | **161.70** |  |

**Table S5** Comparison of the electrochemical performance for AEG and BEG electrodes with the previously reported state-of-the-art graphitic carbon materials.

| **Cathode materials** | **Electrolyte** | **Capacity [mAh g^-1^] /  current density [mA g^-1^]** | **Cycle** | **Potential window [V]** | **Coulombic efficiency  [%]** | **Ref.** |
| --- | --- | --- | --- | --- | --- | --- |
| **Base-etched graphite (BEG)** | **AlCl_3_:[EMIm]Cl**  **= 1.5:1** | **~ 110 / 4,000** | **1,000** | **0.0‑2.45** | **99.9** | **This work** |
|  |  | **~ 91 / 10,000** | **10,000** |  |  |  |
| **Acid-treated expanded graphite (AEG)** |  | **~ 89 / 4,000** | **1,000** |  | **99.1** |  |
|  |  | **~ 80 / 10,000** | **10,000** |  |  |  |
| Graphitic foam | AlCl_3_:[EMIm]Cl  = 1.3:1 | ~ 66 / 66 | 200 | 0.0‑2.5 | ~ 99.3 | [S6] |
|  |  | ~ 60 / 4,000 | 7,500 |  |  |  |
| Mesoporous rGO powder | AlCl_3_:[EMIm]Cl  = 1.3:1 | ~ 150 / 100 | 100 | 0.01**‑**2.2 | ~ 85 | [S7] |
|  |  | ~ 100 / 1,000 | 3,000 |  |  |  |
|  |  | ~ 55 / 10,000 | 25,000 |  |  |  |
| Kish graphite flakes | AlCl_3_:[EMIm]Cl = 1.5:1 | 120 / 500 | 200 | 0.01**‑**2.45 | ~ 80 | [S8] |
| Defect free graphene | AlCl_3_:[EMIm]Cl = 1.3:1 | 100 / 5,000 | 25,000 | 0.7**‑**2.51 | ~ 97 | [S9] |
| Small flake natural graphite | AlCl_3_:[EMIm]Cl = 2:1 | 132 / 100 | 100 | 0.5**‑**2.4 | 92 | [S10] |
| Zeolite-templated carbon | AlCl_3_:[EMIm]Cl = 1.3:1 | ~ 180 / 100 | 500 | 0.01**‑**2.2 | 98–100 | [S11] |
|  |  | ~ 157 / 1,000 | 1,000 |  |  |  |
| Natural graphite flake | AlCl_3_:[EMIm]Cl = 1.3:1 | 100 / 198 | 1,100 | 0.5**‑**2.45 | ~ 99 | [S12] |
|  |  | 60 /660 | 6,000 |  |  |  |
| High purity graphite paper | AlCl_3_:[EMIm]Cl  = 1.3:1 | ~ 70 / 20 | 600 | 0.5**‑**2.4 | ~ 98 | [S13] |
|  |  | ~ 70 / 50 | 200 |  |  |  |

| **Cathode materials** | **Electrolyte** | **Capacity [mAh g^-1^] /  current density [mA g^-1^]** | **Cycle** | **Potential window [V]** | **Coulombic efficiency  [%]** | **Ref.** |
| --- | --- | --- | --- | --- | --- | --- |
| Expanded  graphite | AlCl_3_:[EMIm]Cl  = 1.3:1 | ~ 60 / 1,000 | 3,000 | 0.7**‑**2.51 | 69.8 | [S14] |
|  |  | ~ 60 / 5,000 | 10,000 |  |  |  |
|  | AlCl_3_:ET*  = 1.5:1 | ~ 98 / 1,000 | 5,000 | 0.7**‑**2.54 | 77.5 |  |
|  |  | ~ 78 / 5,000 | 30,000 |  |  |  |
| 3D graphene mesh network | AlCl_3_:[EMIm]Cl  = 1.3:1 | 57 / 240 | 200 | 0.0**‑**2.5 | 97.5 | [S15] |
| Large-sized few-layer graphene | AlCl_3_:[PMIm]Cl  = 1.3:1 | ~ 90 / 60 | 200 | 0.0**‑**2.5 | ~ 95 | [S16] |
|  |  | ~ 80 / 300 | 4,500 |  |  |  |
| Graphite powder | AlCl_3_:[EMIm]Cl  = 1.3:1 | ~ 73 / 100 | 180 | 1.0**‑**2.2 | ~ 99.7 | [S17] |
| Trihigh tricontinuous (3H3C) graphene film | AlCl_3_:[EMIm]Cl  = 1.3:1 | ~ 120 / 6,000 | 16,000 | 0.6**‑**2.5 | ~ 91.7 | [S18] |
|  |  | ~120 / 100,000 | 250,000 |  |  |  |
| Graphite | AlCl_3_:Urea  = 1.5:1 | 50 / 2,000 | 8,000 | 0.3-2.4 | ~95 | [S19] |
| Surface-perforated graphene | AlCl_3_:[EMIm]Cl  = 1.3:1 | ~197 / 2,000 | 200 | 0.5-2.4 | 92.5 | [S20] |
|  |  | ~147 / 5,000 | 1,000 |  |  |  |
| Vein graphite flake | AlCl_3_:[EMIm]Cl  = 2.1:1 | 103 / 100 | 50 | 0.01-2.38 | 80-90 | [S21] |
|  |  | ~90 / 500 | 100 |  |  |  |
| Graphite | AlCl_3_:[EMIm]Cl  = 1.5:1 | ~110 / 200 | 100 | 0.4-2.4 | 88-90 | [S22] |

*ET = triethylamine hydrochloride

**Table S6** Comparison of different technologies (methods) for adequate surface defects and their specific capacities.

| **Cathode materials** | **Technologies** | **Surface defects** | **Specific Capacity /**  **Current rate** | **Ref.** |
| --- | --- | --- | --- | --- |
| **Acid expanded graphite (AEG)** | **Acid (sulfuric/nitric acid) mixing process** | **Expanded the graphitic layers** | **~89 mAh g^-1^ / 4,000 mA g^-1^** | **This work** |
| **Base etched graphite (BEG)** | **Base (4M KOH) etching process** | **Expanded layers and Pores/holes on the graphite surface** | **~110 mAh g^-1^ / 4,000 mA g^-1^** |  |
| Mesoporous rGO powder | Hummer method | Large defect and pores | ~100 mAh g^-1^ / 1,000 mA g^-1^ | [S7] |
| Kish graphite flakes | Sonication | Crater morphology with deep craters(holes) | 120 mAh g^-1^ / 500 mA g^-1^ | [S8] |
| Defect-free graphene | High temperature annealing (>2000℃) | Vacancy holes and polygons | 100 mAh g^-1^ / 5,000 mA g^-1^ | [S9] |
| Zeolite-templated carbon | Impregnation of ion exchanged zeolite Y | High pore-to-pore regularity & high surface area | 157 mAh g^-1^ / 1,000 mA g^-1^ | [S11] |
| Natural graphite flake | Casting and etching | Free-standing surface | 60 mAh g^-1^ / 660 mA g^-1^ | [S12] |
| 3D graphene mesh network | Electroplating and etching | 3D mesh network | 57 mAh g^-1^ / 240 mA g^-1^ | [S15] |
| Trihigh tricontinuous (3H3C) graphene film | High temperature annealing | Honeycomb atomic lattice | ~120 mAh g^-1^ / 6,000 mA g^-1^ | [S18] |
| Vein graphite flake | ultrasonication | Fragmentized particles | 103 mAh g^-1^ / 100 mA g^-1^ | [S21] |
| Graphene nanoribbons porous 3D graphene (GNHPG) | Plasma-etching | Nanovoids distributed on the 3D graphene | 123 mAh g^-1^ / 5,000 mA g^-1^ | [S23] |

**Supplementary References.**

1. K. V. Kravchyk and M. V. Kovalenko, Adv. Energy Mater. **9**, 1901749 (2019).
2. J. Kim, D. Lee, J. Lee, C. Kim, Sci. Rep. **10,** 15586 (2020).
3. Y. Lu, B. M. Gallant, D. G. Kwabi, J. R. Harding, R. R. Mitchell, M. S. Whittinghamd, S. Yang, Energy Environ. Sci. **6**(3), 750 (2013).
4. B. E. Conway, Electrochemical Supercapacitor 417-477 (1999).
5. S. C. Jung, Y.-J. Kang, D.-J. Yoo, J. W. Choi, Y.-K. Han, J. Phys. Chem. C **120**(25), 13384−13389 (2016).
6. M. C. Lin, M. Gong, B. Lu, Y. Wu, D. Y. Wang, M. Guan, M. Angell, C. Chen, J. Yang, B. J. Hwang, H. J. Dai, Nature **520**, 324-328 (2015).
7. J. Smajic, A. Alazmi, N. Batra, T. Palanisamy, D. H. Anjum, P. M. F. J. Costa, Small **14**(51), 1803584 (2018).
8. S. Wang, K. V. Kravchyk, F. Krumeich, M. V. Kovalenko, ACS Appl. Mater. Inter. **9**(34), 28478-28485 (2017).
9. H. Chen, F. Guo, Y. Liu, T. Huang, B. Zheng, N. Ananth, Z. Xu, W. Gao, C. Gao, Adv. Mater. **29**(12) 1605958 (2017).
10. K. V. Kravchyk, S. Wang, L. Piveteau, M. V. Kovalenko, Chem. Mater. **29** (10), 4484-4492 (2017).
11. N. P. Stadie, S. Wang, K. V. Kravchyk, M. V. Kovalenko, ACS Nano **11**(2), 1911-1919 (2017).
12. D. Y. Wang, C. Y. Wei, M. C. Lin, C. J. Pan, H. L. Chou, H. A. Chen, M. Gong, Y. Wu, C. Yuan, M. Angell, Y. J. Hsieh, Y. H. Chen, C. Y. Wen, C. W. Chen, B. J. Hwang, C. C. Chen, H. Dai, Nature Commun. **8**, 14283 (2017).
13. S. Wang, S. Jiao, W. Song, H. Chen, J. Tu, D. Tian, H. Jiao, C. Fu, D. Fang, Energy storage mater. **12**, 119-127 (2018).
14. X. Dong, H. Xu, H. Chen, L Wang, J Wang, W. Fang, C. Chen, M. Salman, Z. Xu, C. Gao, Carbon **148**, 134-140 (2019).
15. G. Y. Yang, L. Chen, P. Jiang, Z. Y. Guo, W. Wang, Z. P. Liu, RSC Adv. **6**(53), 47655-47660 (2016).
16. L. Zhang, L. Chang, H. Luo, X. Zhou, Z. Liu, Adv. Energy Mater. **7**(15), 1700034 (2017).
17. M. Angell, C. Pan, Y. Rong, C. Yuan, M. Lin, B. Hwang, H. Dai, PNAS **14**(5), 834-839 (2017).
18. H. Chen, H. Xu, S. Wang, T. Huang, J. Xi, S. Cai, F. Guo, Z. Xu, W. Gao, C. Gao, Sci. Adv. **3**(12), eaao7233 (2017).
19. F. Jach, M. Wassner, M. Bamberg, E. Brendler, G. Frisch, U. Wunderwald, J. Friedrich, ChemElectroChem **8**, 1988-1992 (2021).
20. Y. Kong, Ch. Tang, X. Huang, A.K. Nanjundan, J. Zou, A. Du, C. Yu, Adv. Funct. Mater. **31**, 2010569 (2021).
21. S. Wang, M.V. Kovalenko, K.V. Kravchyk, Batter. Supercaps. **4**, 929-933 (2021).
22. G.A. Elia, K. Hoeppner, R. Hahn, Batter. Supercaps. **4**, 368-373 (2021).
23. X. Yu, B. Wang, D. Gong, Z. Xu, B. Lu, Adv. Mater. **29**(4), 1604118 (2017).
